# How to optimize dust pollution control in opencast coal mines: Analysis of a joint social regulation model based on evolutionary game theory

**DOI:** 10.1371/journal.pone.0289164

**Published:** 2023-07-26

**Authors:** Xu Lian, Wensheng Wang, Jianmin Zhang

**Affiliations:** 1 School of Management, China University of Mining and Technology-Beijing, Beijing, China; 2 Research Institute of Decision-making Science and Big Data, China University of Mining and Technology-Beijing, Beijing, China; Chongqing University, CHINA

## Abstract

The carbon peaking and carbon neutrality goals drive innovation in pollution governance systems, unleashing the potential of social supervisory forces to achieve coordinated governance by multiple stakeholders. In order to improve dust pollution control in opencast coal mines, this study combines prospect theory with evolutionary game theory, analyzing the evolutionary game process of coordinated governance activities of coal mining enterprises, local regulators, and social camps in the management of dust pollution against the backdrop of national supervisions. The research indicates that the perceived value of dust pollution has a significant impact on the strategic choices of the three agents involved in the game. Coal mining enterprises tend to be risk averse, and by reducing the cost of dust pollution control and increasing the additional benefits of pollution control, it can promote pollution control behavior by coal mining enterprises. Local regulators are also risk averse, but not sensitive to risk benefits. Strengthening pollution subsidy incentives and environmental fines can help promote dust pollution control behavior by coal mining enterprises. However, increasing the strength of the rewards strategy is not conducive to local regulators’ own regulatory responsibilities, and environmental fines have limited binding effects. The strategic choices of social camps’ supervision have a restrictive effect on the strategic choices of coal mining enterprises and local regulators, promoting the evolution of equilibrium results in the direction of maximizing social benefits. When coal mining enterprises actively governance pollution, local regulators strictly regulated, and social camps do not monitor, the system reaches its optimal equilibrium state. The research results clarify the mechanism and specific effects of social supervision of opencast coal mine dust pollution control, guide the participation of the public in dust pollution control, and regulate the behavior strategies of coal mining enterprises and local regulators, providing the scientific basis for management.

## Introduction

Coal is the key energy source for industrial development. For a long time, China’s economic and social development has had a strong demand for coal, and the coal industry has developed rapidly [1, 2]. Compared with other types of mines, opencast mines have higher safety levels, higher coal recovery rates, larger production scales, higher mechanization levels, and higher production efficiency, so they are given priority for construction, and the number and proportion continue to increase. However, the design and construction ideas for opencast mines are overly conservative, and the attention to dust pollution control is very low, resulting in dust pollution control issues in opencast coal mining enterprises not being given adequate attention for a long time. According to national statistical investigation, it has been found that the average dust concentration in China’s opencast coal mines exceeds the standard by 5 to 43 times, with a dust pass rate of only 13%. The pollution caused by dust has worsened the working environment of coal mining and transportation, and posed a potential threat to the physical well-being of workers in these opencast mines along with the surrounding communities [3, 4]. The 20th National Congress of the Communist Party of China proposes to further advance environmental pollution prevention and control, adhering to precise, scientific, and lawful methods. At present, dust pollution in China’s opencast coal mines has become a prominent issue, obstructing the development of the nation’s green and ecological civilization construction, as well as the achievement of the carbon peaking and carbon neutrality goals. As the backbone of dust pollution control, coal mining enterprises need to be better regulated and guided to implement the concept of green development actively and thoroughly, internalizing pollution externalities for quality improvement of the ecological environment in mining areas. To achieve this, optimization and upgrading of the dust pollution control model is required, to promote the development of a green, low-carbon and circular economy [5].

China employs a vertical governance system for environmental pollution control, which follows a top-down "unified" approach. The Ministry of Ecology and Environment of the People’s Republic of China serves as the central institution that formulates comprehensive policies and assumes the supervisory role. The actual task of pollution supervision is undertaken by local ecological and environmental departments [6]. The excessive length of the functional chain often leads to the problem of information asymmetry. It is difficult to identify and control illegal behaviors such as fraud and collusion between coal mining enterprises and local regulators in pollution control work. As a result, pollution control policies are unable to play their due role effectively [7]. Indeed, due to factors such as regulatory costs and technical thresholds, local environmental protection bureaus can only carry out pollution control through periodic and sudden inspections, which cannot provide continuous and ubiquitous monitoring of the mining area environment, resulting in problems such as untimely regulation and monitoring blind spots. Some opencast coal mining enterprises are only interested in profits, lacking firm environmental protection awareness and social responsibility, which exacerbates the situation [8]. As the most direct victims of coal mining dust pollution, residents in mining areas suffer greatly from illegal discharge of pollutants. However, their reports to local regulators often go unheeded, and government regulation proves ineffective. Consequently, nimby conflicts arise frequently due to failures of regulation [9]. Based on the above analysis, the current system of coal mining dust pollution control in opencast mines in China still suffers from problems such as incomplete regulations and insufficient regulatory efforts, resulting in a lack of effective improvement in mining area environments.

As the concept of sustainable, green development becomes increasingly popular, public awareness of environmental protection and public scrutiny also rise. The news media, public, and third-party organizations have become important social forces in supervising the regulation of coal mining dust pollution. The use of internet platforms to participate in environmental pollution control has been effective, as online public opinion has amplified the negative impact of coal mining dust pollution, putting pressure on coal mining enterprises and government regulators to act. This has had a positive effect on urging coal mining enterprises to improve their dust pollution control measures and on government regulators to increase monitoring efforts [10]. Therefore, in the process of controlling dust pollution in open coal mines, introducing and activating social forces to promote the transformation of the dust pollution control system from "unitary" governance to "diverse" governance, creating a collaborative supervision mode for dust pollution with "government leadership, corporate management and social supervision", and achieving maximum social benefits, are important measures for managing dust pollution in opencast coal mines [11, 12].

Exploring the interaction between stakeholders has been the focus of environmental governance research. How to promote the regulators to effectively regulate the illegal discharges behavior of enterprises, game theory is widely used to study this problem. Zhang et al. [13] established a tripartite evolutionary game model between the central government, local governments, and polluting enterprises to study the mechanism of how central environmental inspections influence the management of atmospheric pollution, and believed that central environmental inspectors can be effective in encouraging polluting companies to clean up their act, but their incentive effect on local government regulatory strategies needs to be further improved. Gao and Xi [14] built a two-party evolutionary game matrix between the government and wastewater-polluting enterprises, and found that governments can develop dynamic bilateral strategies based on the behavior of polluting companies. To explore the governance mechanism of diversified co-governance among the government, polluting enterprises, and third-party governance enterprises in the treatment of soil heavy metal dust pollution, Zhou et al. [15] believed government can curb the illegal behavior of polluting enterprises through administrative control, and third-party governance of enterprises will change the rational expectations of polluting enterprises. Wang et al. [16] set up a dual subject evolutionary game model between local governments and rare earth enterprises, and propose relevant suggestions for the treatment of dust pollution in rare earth mining areas.

As the evolutionary game-related assumptions do not consider the impact of decision-making agents on decisions, some scholars have incorporated prospect theory into evolutionary game models. Prospect theory considers non-rational factors, such as risk preferences and cognitive differences of decision-making agents, in the decision-making process, thereby changing decision-making based on rational calculations of risks and returns and breaking through the rational expected benefits hypothesis. As a result, evolutionary game research is now closer to real-world situations [17]. Combining the evolutionary game model of prospect theory, Gao et al. [18] studied the environmental regulation strategies to control dust pollution in marine ecology and found that there are differences in the risk sensitivity of different environmental governance entities. In the context of social organizations and public participation, Xu [19] examined the behavioral interactions between local governments and enterprises and their influencing factors, while Qi [20] set up a game model for both agents and demonstrates that the involvement of social organizations and the public in environmental governance can effectively stimulate the government’s regulatory efforts, and believed that the development of appropriate strategies on the basis of risk sensitivity will be more targeted and therefore produce better results. Tan and Xu [21] constructed an evolutionary game model for environmental pollution enterprises and the local people using the prospect value function. The conclusion of the research showed that environmental companies and the people around them have the characteristics of income perception and loss aversion. Shen et al. [22] studied local governments and pollution enterprises in a river basin and analyzed them through a two-player game model that incorporates prospect theory to solve cross-regional water pollution problems and found that the marginal decreasing degree of value function has a stronger influence on local governments than on polluting enterprises. By constructing a three-player evolutionary game model that incorporates prospect theory, Xu et al. [23] proposed suggestions for the effective management of inland waterway pollution to achieve a navigational governance. He et al. [24] explored the game strategy selection mechanism among the government, social capital, and the public in major engineering projects.

The above literature confirms the positive role of the government in environmental governance, but in practical regulatory situations, due to conflicts of interest, enterprises and the government may have conflicts of interest and make different strategic choices, greatly reducing the efficiency of government regulation, and need to joint other forces to create a diversified governance model and improve environmental governance efficiency. In addition, most studies have only indirectly addressed the other types of pollution control under general concepts, without providing feasible solutions for dust pollution control in opencast coal mines. There remains a dearth of studies focusing on the mechanisms for controlling dust pollution in opencast coal mines, as well as a lack of practical and effective dust suppression methods.

Therefore, in order to fill these research gaps, this study takes the dust pollution control of opencast coal mines as the research issue, combines prospect theory and evolutionary game theory, establishes a game model among coal mining enterprises, local regulators, and social camps. The main contributions are as follows: At first, this study illustrates China’s environmental protection system and the situation of dust pollution in opencast coal mines, addresses the challenges of dust pollution, which is characterized by high carbon emissions, high harm, and complexity in opencast coal mines, and shows the necessity and urgency of building a multi-agent collaborative governance system. Then, this study introduces social monitoring to compensate for the shortcomings of traditional national environmental management. Combining prospect theory and evolutionary game theory, a tripartite game model was developed for coal mining enterprises, local regulators, and the social camps. Next, by solving the model, this study investigates the decision-making behaviors of changing the three parties in the pollution control of opencast coal mines. At last, combined with numerical simulation technology, the main factors influencing their decision-making behaviors were analyzed. This enriches the strategy design for the control of dust pollution in opencast coal mines.

In summary, the innovation points of this study can be categorized into the following four aspects: Firstly, from the point of view of the progress of the times, the inclusion of the social camps in the analytical framework of environmental governance is of great importance for improving the traditional system of environmental governance, which focuses only on the government. Secondly, by using the value function and decision weight function in prospect theory to modify the traditional evolutionary game matrix, this study expands the discussion of psychological factors such as risk attitude and perceived value of loss-win in the research on the regulation of dust pollution in opencast coal mines. Finally, this study explores the changing process and key influencing factors of game-based tripartite decision-making behaviors in the control of dust pollution in opencast coal mines Finally, this article discuss the impact of different scenarios and parameters on the cooperative governance mode of dust pollution in opencast coal mines, corresponding optimization measures and tool innovations are proposed to promote the transformation and upgrading of the dust pollution control system in opencast coal mines under the carbon peaking and carbon neutrality goals, which can provide a more comprehensive collaborative governance mechanism and an important reference for relevant policy makers.

## Multi-agent game analysis of the co-regulation model

### Theoretical framework

Different stakeholders have different demands and interests. The issue of dust pollution in opencast coal mines involves numerous stakeholders. Depending on their respective interests, game players can be classified as coal mining enterprises, local regulators, and social camps. To simplify the model, residents around the coal mining enterprises, non-governmental organizations such as environmental protection and healthcare, and media outlets are collectively referred to as the social camps [25]. Coal mining enterprises focus on maximizing their own economic interests and often ignore their social responsibility for environmental protection, given the high cost of dust pollution control. Meanwhile, local regulators may act as rational economic agents and collude with coal mining enterprises to disregard social demands for public interests, leading to a significant reduction in the effectiveness of dust pollution regulation. The social camps are the most direct perceivers of the effectiveness of dust pollution control, yet their participation in such control measures is not very high due to the lack of sufficient protection and funding. When suffering from coal mine dust pollution damage, they often choose to remain silent and endure the damages because of the risks involved in safeguarding their legitimate rights and interests.

Evolutionary game theory integrates the ideas of rational economics and evolutionary biology. Methodologically, it differs from game theory in its emphasis on bounded rationality, its focus on static equilibrium and comparative static equilibrium, and its emphasis on dynamic equilibrium [26]. Evolutionary game theory is an important analytical tool for studying the interaction between multiple agents. In addition, prospect theory is a field of research in psychology that analyses human judgements and decisions under uncertainty. The subjective utility function pursued by each individual and the subjective probability of different possibilities occurring are different, leading to individual differences in judgement and decision making. Based on practical research, the development process of the co-regulation model for dust pollution control in opencast coal mines requires the participation of three stakeholders: the coal mine enterprises, the local regulators, and the social camps. Firstly, there is a mutual interest behavior among coal mining companies, local government regulatory departments and social camps, and their behavioral strategies will affect the strategic choices of other entities. Secondly, the complexity and differences of many factors in the decision-making process, such as capital, risk, and social image, have a certain impact on the psychological factors of each subject, which determine the bounded rationality of the subject’s choice and judgement. Finally, as the external environment of resources, environment, economy and society changes, the decision-making behaviors of stakeholders in dust pollution will also undergo dynamic changes. To sum up, the construction of a co-regulation model for opencast coal mine dust pollution is the result of stakeholders’ continuous interactive adjustment under the influence of different rules and scenarios, and finally reach a dynamic equilibrium, which is consistent with the theoretical connotation of evolutionary game theory and prospect theory. Therefore, in order to simulate the dynamic game process of coordinated control of dust pollution more accurately in opencast coal mines, this study uses evolutionary game theory combined with prospect theory to conduct research.

**Fig 1:**
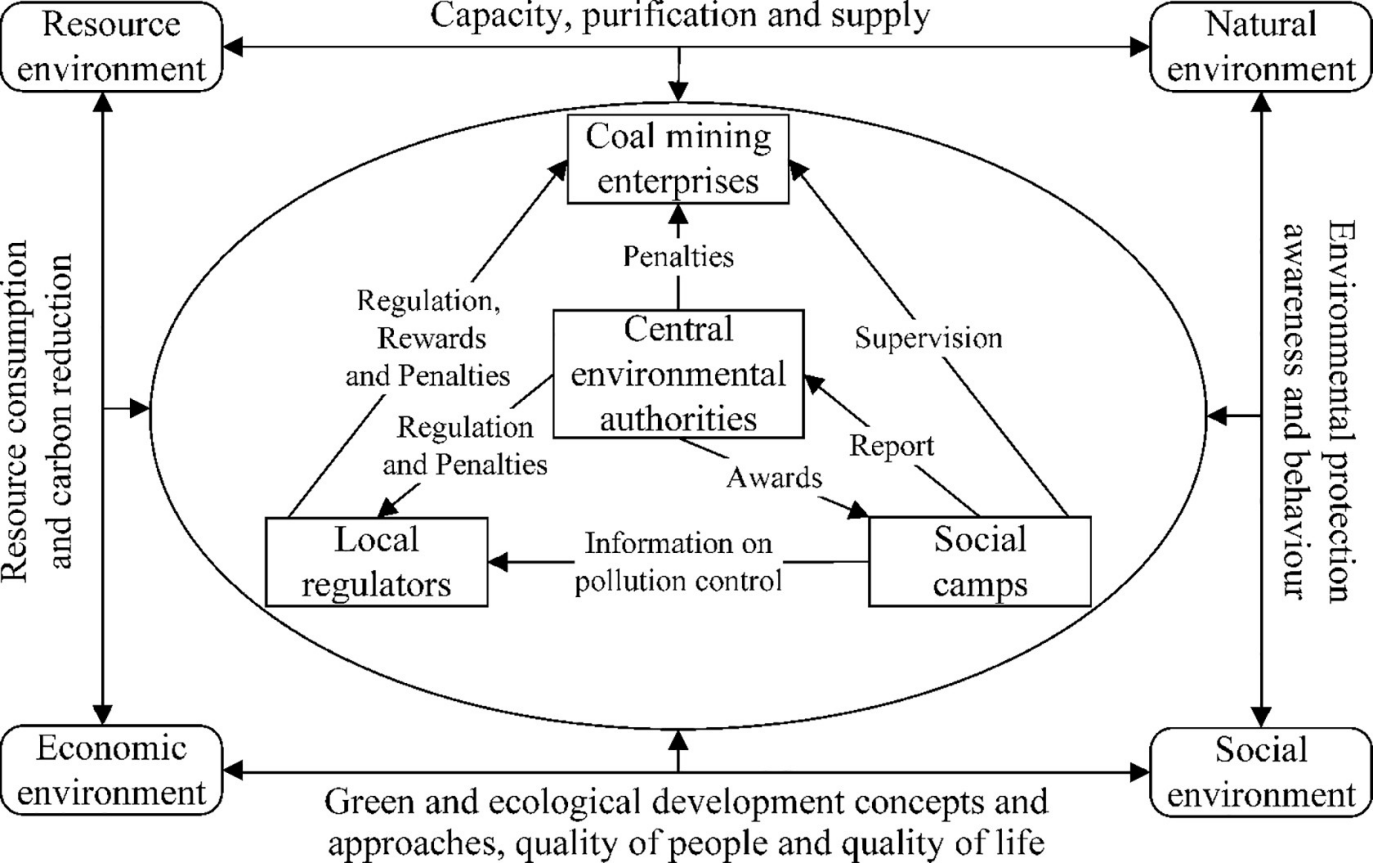
Subject relationship in the co-regulation model for opencast coal mine dust pollution.

Based on the above analysis, the coal mine dust pollution co-regulation model introduces the social camp’s monitoring over coal mining enterprises and expands the scope of pollution regulation. By reporting to and rewarding the central environmental protection department, the effectiveness of complaints and reports is improved, and the enthusiasm for social camps monitoring is enhanced, thereby increasing the strength of pollution regulation, and achieving collaborative regulation by multiple entities. The aim is to avoid or reduce the problem of low regulatory efficiency caused by collusion between officials and enterprises, with the aim of reducing dust pollution, improving environmental ecological quality, and ensuring people’s physical and mental health. The multi-agents game relationship involved in the co-regulation of dust pollution in opencast coal mines is shown in Fig 1.

Assuming that the synergistic regulation of opencast coal mine dust pollution is viewed as a game process between coal mining enterprises, local regulators, and the social camps, without considering the influence of adjacent regions and other entities, the game result directly affects the effect of coal mine dust pollution control. Based on the tripartite evolutionary game system, the following assumptions are made.

### Game hypothesis and design

#### Hypothesis 1

**(H1)** In the multi-agent game process of synergistic regulation of coal mine dust pollution, there exist three types of roles: coal mining enterprises, local regulators, and the social camps. All three agents have limited rationality, based on the principle of profit maximization, choose a predetermined behavior to repeatedly play against other roles under incomplete information. Game strategies are constantly adjusted and optimized until the optimal behavioral strategy that satisfies the interests of game agents is achieved, thereby realizing the equilibrium of the evolutionary game system. Additionally, the enforcement capabilities of the central environmental protection department are sufficiently strong. Once reported and supervised, any violations such as coal mine dust pollution can be accurately identified, and local regulators and coal mining enterprises cannot evade any punishment by any means.

#### Hypothesis 2

**(H2)** To better align the model with the actual process, we can adjust the game decision of the limited rational players by introducing prospect theory. This considers the impact of subjective psychological perception changes of game roles on the formulation of behavioral strategies. Specifically, under the condition of risk aversion, the formulation of game strategies by game roles depends on subjective evaluations and perceived values of events, rather than the actual results themselves [27].

When devising their strategies within the game, agents establish a value reference point ω_0_ based on their inherent characteristics. Subsequently, their decision-making process is determined based on whether the decision result falls greater or lower than this reference point. The perceived benefits of game strategies selection, denoted by V, derive from a joint decision of a weight function π(p_i_), reflecting the likelihood of event p_i_ occurring, and the value function v(Δω_i_), measuring the change in payoffs relative to the reference point. Specifically, the benefits of V derive from the following formula: V = ∑_i_ π(p_i_)v(Δω_i_). The formulae for the weighting function π(p_i_) and the value function v(Δω_i_) are given below.


$$\pi (p_{i})=\frac{p_{i}{}^{\delta }}{{\left[p_{i}{}^{\delta }+{\left(1-p_{i}\right)}^{\delta }\right]}^{\frac{1}{\delta }}}$$ (1)

$$v(\Delta \omega_{i})=\{\begin{array}{l} {\left(\Delta \omega_{i}\right)}^{\alpha },\Delta \omega_{i}\geq 0\\ -\lambda {\left(-\Delta \omega_{i}\right)}^{\beta },\Delta \omega_{i}<0 \end{array}$$ (2)

In Formula 1, p_i_ represents the probability of event i occurring, while δ represents the adjustment coefficient. Based on the premise of the prospect theory, it is assumed that game agents tend to underestimate high probability events and overestimate low probability events, i.e. π(1) = 1 and π(0) = 0 [28].

In Formula 2, α and β represent risk attitude coefficients (0<α, β<1), where higher values indicate a greater preference for taking risks. λ represents the loss aversion coefficient (λ>1), reflecting the degree to which game agents are more sensitive to potential losses than gains. In current research combining prospect theory and evolutionary game theory, the focus is on analyzing the impact of decision makers’ risk preferences on strategy selection. To facilitate analysis, researchers often set the value reference point to zero (ω_0_ = 0), with the difference in values for each option (i.e., Δω_0_ = ω_i_-ω_0_ = ω_i_), simply equaling the value of the option itself ω_i_.

#### Hypothesis 3

**(H3)** Assuming that the strategies adopted by coal mining enterprises are {Active governance, Illegal discharge}, let x(0≤x≤1) represent the proportion of dust pollution control conducted by coal mining enterprises. When x = 1, coal mining enterprises utilize an active governance strategy by duly enforcing environmental policies, proactively assuming environmental and social responsibilities, applying new technologies, equipment, and processes to reduce dust pollution in coal mines, thus effectively improving the mining environment and safeguarding the health of employees and surrounding residents. C_m_ denotes the input costs for dust control equipment purchases, technology research and development, infrastructure construction, and employee training cost. S represents the reward for dust pollution control issued by local regulators. R_m_ represents the additional benefits brought by the improvement of resource exploration and utilization efficiency, the comprehensive utilization and resource utilization of coal powder and waste, and the enhancement of brand image and talent benefits brought by "clean coal". On the other hand, when x = 0, coal mining enterprises adopt an illegal strategy that wreaks negative impact on local economic development, and in the event of being detected by local regulators, P_m_ fines are to be paid.

#### Hypothesis 4

**(H4)** Assuming that the strategies adopted by local regulators are {Strictly regulated, Negligence}, let y(0≤y≤1) represent the proportion of stringent supervision conducted by local regulators. When y = 1, local regulators adopt strict supervision measures by implementing policies and regulations, taking measures to combat coal mine dust pollution, and performing their due diligence in supervision. The cost of strict supervision is denoted as C_l_. When y = 0, local regulators adopt a dereliction of duty strategy, exhibiting weaker supervisory efforts and tolerating or even passively endorsing coal mine dust pollution. As a result of the failure of local regulators to fulfill their duties, coal mine dust pollution continues to affect the normal lives of people in nearby areas. If the situation is reported to the superior environmental protection department and the central government, the local regulators and coal mining enterprises will jointly bear the cost of pollution treatment and government credibility loss, which is denoted by E, and these costs will be apportioned based on a responsibility sharing coefficient γ(0≤γ≤1).

#### Hypothesis 5

**(H5)** Assuming that the strategies adopted by social camps are {Proactive monitoring, No monitoring} so, let z(0≤z≤1) represent the proportion of active supervision conducted by social camps. When z = 1 social camps adopt active monitoring measures, utilizing national inspection agencies’ internet platforms or news media to supervise and report on coal mining enterprises’ illegal emissions and collusive activities with government officials. The cost of monitoring is denoted as C_s_ The national supervision agency checks and provides feedback on reported information, and if the information is verified to be true, rewards of R_s_ are issued. As per the actual situation, the profit derived from active supervision by social camps is positive, that is, it satisfies the condition R_s_>C_s_. When z = 0, social camps adopt a strategy of not monitoring, meaning they do not participate in coal mine dust pollution supervision and may choose to remain silent and tolerate damage caused by pollution.

Based on the previous text, the relevant parameters, and descriptions of the evolutionary game model for the co-regulation of opencast coal mine dust pollution are shown in Table 1.

**Table 1:** Interpretation of the parameters of the evolutionary game model.

Game agent	Parameters	Definition
**Coal mining enterprises**	x	Probability of coal mining enterprises actively combating dust pollution
	C_m_	Pollution control costs
	P_m_	Environmental fines for illegal discharges
	R_m_	Additional benefits of pollution control
	S	Subsidies for environmental protection
	β_m_	Risk pursuit factor for coal mining enterprises
	λ_m_	Loss aversion factor for coal mining enterprises
**Local regulators**	y	Probability of strict regulation by local regulators
	C_l_	Pollution control costs
	γ	Responsibility sharing coefficient
	α_l_, β_l_	Risk pursuit factor for local regulators
	λ_l_	Loss avoidance factor for local regulators
**Social camps**	z	Probability of proactive monitoring by the social camps
	C_s_	Active monitoring costs
	R_s_	Reward for successful pollution reporting
	α_s_	Social camps risk pursuit factor
**Responsible party for pollution**	E	Covering expenses such as negative externalities and loss of credibility caused by dust pollution

Based on the assumptions, a tripartite game revenue matrix for co-regulation of dust pollution in opencast coal mines can be constructed, as shown in Table 2.

**Table 2:** Evolutionary tripartite benefit matrix.

Three agents in the game		Local regulators	Social camps	
			Proactive monitoring	No monitoring
**Coal mining enterprises**	Active governance	Strictly regulated	-C_m_+S+R_m_ -C_l_ -S -C_s_	-C_m_+S+R_m_ -C_l_ -S 0
		Negligence	-C_m_+R_m_ V(-γE) -C_s_	-C_m_+R_m_ V(-γE) 0
	Illegal discharge	Strictly regulated	-P_m_-E -C_l_+P_m_ -C_s_	-P_m_-E -C_l_+P_m_ V (R_s_ -C_s_)
		Negligence	-(1-γ) E -γE+V (P_m_) R_s_ -C_s_	V [-(1-γ) E] V (-γE) +V (P_m_) V (R_s_-C_s_)

### Game model solution

Based on the assumptions and game model, establish the replicator dynamic equation and equilibrium solution, and analyze the evolutionary game process of the three agents: coal mining enterprises, local regulators, and social camps.

Let E_x1_ denote the expected return of coal enterprises choosing the {Active governance} strategy, E_x2_ denote the expected return of coal enterprises choosing the {Illegal discharge} strategy, and $\bar{E_{x}}$ denote the average expected return of the two strategies of coal enterprises, then:

$$\left\{ \begin{array}{l} E_{x1}=yz(-C_{m}+S+R_{m})+(1-y)z(-C_{m}+R_{m})+y(1-z)(-C_{m}+S+R_{m})\\ \;+(1-y)(1-z)(-C_{m}+R_{m})\\ \;=R_{m}-C_{m}+yS\\ E_{x2}=yz(-P_{m}-E)+(1-y)z[-(1-\gamma )E]+y(1-z)(-P_{m}-E)\\ \;+(1-y)(1-z)[V(-(1-\gamma )E)]\\ \;=(y-1)z[(1-\gamma )E]-y(E+P_{m})\\ \;+(y-1)(z-1)[V(-(1-\gamma )E)]\\ \bar{E_{x}}=xE_{x1}+(1-x)E_{x2} \end{array}\right.$$ (3)

Similarly, the set of equations for the expected benefits for the local regulators and the social camps, respectively, are:

$$\left\{ \begin{array}{l} E_{y1}=xz(-C_{l}-S)+(1-x)z(-C_{l}+P_{m})+x(1-z)(-C_{l}-S)\\ \;+(1-x)(1-z)(-C_{l}+P_{m})\\ \;=(1-x)P_{m}-C_{l}-xS\\ E_{y2}=xz[V(-\gamma E)]+(1-x)z[-\gamma E+V(P_{m})]+x(1-z)[V(-\gamma E)]\\ \;+(1-x)(1-z)[V(-\gamma E)+V(P_{m})]\\ \;=(x-1)(z-1)[V(-\gamma E)+V(P_{m})]-(x-1)z[-\gamma E+V(P_{m})]\\ \;+xV(-\gamma E)\\ \bar{E_{y}}=yE_{y1}+(1-y)E_{y2} \end{array}\right.$$ (4)

And,

$$\left\{ \begin{array}{l} E_{z1}=xy(-C_{s})+(1-x)y(-C_{s})\\ \;+x(1-y)(-C_{s})+(1-x)(1-y)(R_{s}-C_{s})\\ \;=R_{s}(1+xy-x-y)-C_{s}\\ E_{z2}=xy*0+(1-x)y[V(R_{s}-C_{s})]+x(1-y)*0\\ \;+(1-x)(1-y)[V(R_{s}-C_{s})]\\ \;=-(x-1)[V(R_{s}-C_{s})]\\ \bar{E_{z}}=zE_{z1}+(1-z)E_{z2} \end{array}\right.$$ (5)

In the above equation, E_y1_ denotes the expected return of the local regulator choosing the {Strict supervision} strategy, E_y2_ denotes the expected return of the local regulator choosing the {Negligence} strategy, $\bar{E_{y}}$ denotes the average expected return of the two strategies of the local regulators. E_z1_ denotes the expected return of the social camp choosing the {Proactive monitoring} strategy, E_z2_ denotes the expected return of the social camp choosing the {No monitoring} strategy. $\bar{E_{z}}$indicates the average expected return of the two strategies in the social camps.

According to Friedman’s replicator dynamic model, the replicator dynamic equation reflects the adjustment and variation of the proportion of dominant strategies chosen by the game entities [29]. The replicator dynamic equations for the three-agent game among coal mining enterprises, local regulators, and social camps are as follows:

$$\begin{array}{l} F_{CM}(x)=\frac{dx}{dt}=x(E_{x1}-\bar{E_{x}})=x(1-x)(E_{x1}-E_{x2})\\ \;=x(x-1)\left\{\begin{array}{l} C_{m}-R_{m}-y(E+P_{m}+S)+(y-1)z[(1-\gamma )E]\\ +(y-1)(z-1)[V[-(1-\gamma )E]] \end{array}\right\} \end{array}$$ (6)

$$\begin{array}{l} F_{LG}(y)=\frac{dy}{dt}=y(E_{y1}-\bar{E_{y}})=y(1-y)(E_{y1}-E_{y2})\\ \;=y(y-1)\left\{\begin{array}{l} C_{l}+(x-1)P_{m}+xS+xV(-\gamma E)\\ +(x-1)z[\gamma E-V(P_{m})]\\ +(x-1)(z-1)[V(-\gamma E)+V(P_{m})] \end{array}\right\} \end{array}$$ (7)

$$\begin{array}{l} F_{SC}(z)=\frac{dz}{dt}=z(E_{z1}-\bar{E_{z}})=z(1-z)(E_{z1}-E_{z2})\\ \;=z(z-1)[C_{s}+R_{s}(x+y-xy-1)-(x-1)V(R_{s}-C_{s})] \end{array}$$ (8)

Of which, each prospect theory function of the relevant game agents are as follows:

$$\left\{ \begin{array}{l} V[-(1-\gamma )E]=-\lambda_{m}{\left(-\gamma E\right)}^{\beta_{m}}\\ V(-\gamma E)=-\lambda_{l}{\left(-\gamma E\right)}^{\beta_{l}}\\ V(P_{m})=P_{m}{}^{\alpha_{l}}\\ V(R_{s}-C_{s})={\left(R_{s}-C_{s}\right)}^{\alpha_{s}} \end{array}\right.$$ (9)

The replicator dynamic equation reveals that the variation in the selection proportions of game strategies by the game players is influenced by the game payoffs.

### Evolutionary stability analysis

#### Single-agent stability analysis

(1) The analysis for coal mining enterprises

According to the stability theorem of differential equations, to determine whether the strategy results of coal mining enterprises is in a stable state, it is necessary to simultaneously satisfy F_CM_(x) = 0 and ∂F_CM_(x)/∂x<0, that is:

$$\frac{\partial F_{CM}(x)}{\partial x}=(2x-1)\{\begin{array}{l} C_{m}-R_{m}-yE-yP_{m}-yS\\ -(1-y)z(1-\gamma )E+(y+z-yz-1)\lambda_{m}[(1-\gamma )E^{\beta_{m}}] \end{array}\}$$ (10)

Suppose that M(y) is a function of y.


$$M(y)=\{\begin{array}{l} C_{m}-R_{m}-yE-yP_{m}-yS-\\ \left(1-y)z(1-\gamma )E+(y+z-yz-1)\lambda_{m}[(1-\gamma )E^{\beta_{m}}\right] \end{array}$$ (11)

$$y^{*}=\frac{C_{m}-R_{m}-z(1-\gamma )E+(z-1)\lambda_{m}[(1-\gamma )E^{\beta_{m}}]}{E+P_{m}+S+z(1-\gamma )E-(z-1)\lambda_{m}[(1-\gamma )E^{\beta_{m}}]}$$ (12)

**Fig 2:**
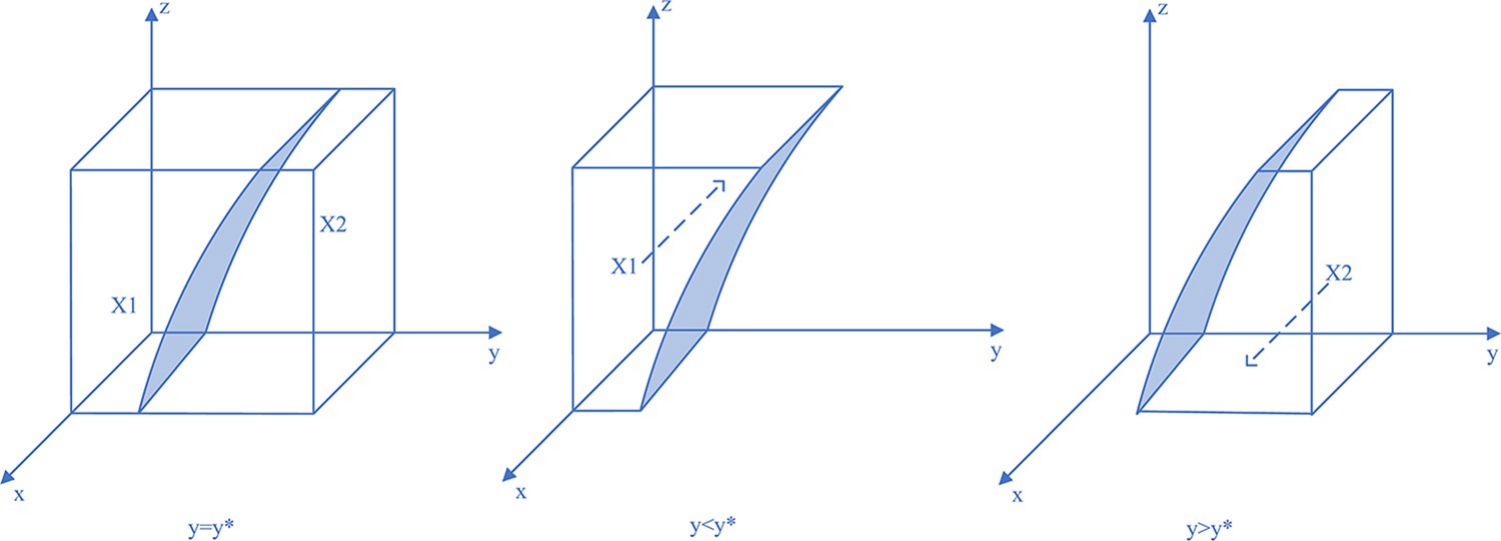
Coal mining enterprises strategy replication dynamic phase diagram.

Therefore, when y = y*, M(y) = 0, and ∂F_CM_(x)/∂x≡0, the system can reach a steady state regardless of the value of *y*, and the coal mining enterprises have no evolutionary stabilization strategy; if y<y*, M(y)>0, and ∂F_CM_(x)/∂x|_x = 0_<0, at which point x = 0, the evolutionary stabilization strategy of the coal mining enterprises is illegal discharge. Similarly, and conversely, for x = 1, the coal mining enterprises’ evolutionary stabilization strategy is active governance. The replicated dynamic phase diagram of the coal mining enterprises’ strategy is shown in Fig 2.

**Inference 1**: The probability of coal mining enterprises actively managing dust pollution is positively correlated with the additional benefits of dust pollution control, pollution compensation expenses, pollution fines, governance subsidies, risk-pursuit coefficients, and loss-avoidance coefficients, and is negatively correlated with governance costs and responsibility-sharing coefficients. It follows that coal mining enterprises should adopt green dust suppression technologies to reduce the input costs of traditional dust pollution control such as water resources and road maintenance, and increase the benefits of dust pollution control such as improving operational environment and safety measures. This can effectively prevent illegal pollution discharge from coal mining enterprises. Local regulators can, on the one hand, constrain illegal pollution discharge from coal mining enterprises by increasing reward and punishment measures, and on the other hand, mitigate the post-discharge compensation expenses and loss of credibility of the offending enterprises by establishing effective information disclosure systems and enhancing environmental and cultural information dissemination, thereby promoting the active management of dust pollution by coal mining enterprises.

**Inference 2**: The probability of coal mining enterprises engaging in dust pollution control behavior is influenced by the strategic choices of local regulators and social camps. Under the condition 0<y*<1, coal mining enterprises will gradually evolve towards a proactive governance strategy when local regulators strictly regulate and social camps actively supervise. When local regulators neglect their duties and social camps do not monitor, coal mining enterprises will gradually evolve into a strategy of illegal pollution discharge. At the same time, the lower y* is, the more effective the supervision strategies of local regulators and social camps will be in promoting coal mining enterprises to develop towards proactive governance.

(2) The analysis for local regulators

According to the stability theorem of differential equations, to determine whether the strategy results of local regulators is in a stable state, it is necessary to simultaneously satisfy F_LG_(y) = 0 and ∂F_LG_(y)/∂y<0, that is:

$$\frac{\partial F_{LG}(y)}{\partial y}=(2y-1)[\begin{array}{l} C_{l}-(1-x)P_{m}{}^{\alpha_{l}}+xS-\lambda_{l}{\left(\gamma E\right)}^{\beta_{l}}\\ -(1-x)z\gamma E+(1-x)z\lambda_{l}{\left(\gamma E\right)}^{\beta_{l}} \end{array}]$$ (13)

Suppose that L(z) is a function of z.


$$L(z)=C_{l}-(1-x)P_{m}{}^{\alpha_{l}}+xS-\lambda_{l}{\left(\gamma E\right)}^{\beta_{l}}-(1-x)z\gamma E+(1-x)z\lambda_{l}{\left(\gamma E\right)}^{\beta_{l}}$$ (14)

$$z^{*}=\frac{C_{l}-(1-x)P_{m}{}^{\alpha_{l}}+xS-\lambda_{l}{\left(\gamma E\right)}^{\beta_{l}}}{(1-x)\gamma E-(1-x)\lambda_{l}{\left(\gamma E\right)}^{\beta_{l}}}$$ (15)

**Fig 3:**
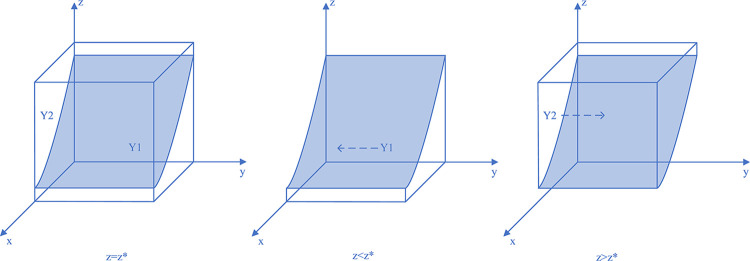
Local regulators strategy replication dynamic phase diagram.

Thus, when z = z*, L(z) = 0, and ∂F_LG_(y)/∂y≡0, the system reaches a steady state regardless of the value of z and the local regulator has no evolutionary stabilization strategy; if z<z*, G(z)>0, ∂F_LG_(y)/∂y|_y = 0_<0, at which point y = 0, the evolutionary stabilization strategy of the local regulators is negligence. Similarly, and conversely, for y = 1, the evolutionary stabilization strategy of the local regulators is strictly regulated. The replicated dynamic phase diagram of the local regulators’ strategy is shown in Fig 3.

**Inference 3**: The probability of local regulators strictly regulating is positively correlated with pollution fines, risk-pursuit coefficients, risk-avoidance coefficients, responsibility-sharing coefficients, and pollution compensation expenses, and negatively correlated with pollution subsidies and regulatory costs. It follows that the increase in pollution penalties should be based on the pollution control costs of coal mining enterprises and their awareness of dust pollution, while strengthening publicity and education on dust pollution risks can increase the public’s awareness of the issue, which can help local regulators evolve their strategic choices towards strict monitoring. Additionally, an appropriate increase in the regulatory budget can prevent local regulators from engaging in "sloth administration" behaviors to a certain extent.

**Inference 4:** The positive strategies of coal mining enterprises and social camps can lead local regulators to evolve towards stricter monitoring. Under the condition 0<z*<1, as coal mining enterprises engage in illegal pollution discharge and social camps actively supervise, the strategies of local regulators will gradually evolve towards proactive governance. Conversely, when coal mining enterprises actively engage in pollution control and social camps do not monitor, the strategies of local regulators may gradually devolve towards neglecting their duties. This also indicates that social camps have a supervisory role in the performance of local regulators. Additionally, the lower z* is, the more effective the positive strategies of coal mining enterprises and social camps will be in promoting the development of local regulators towards stricter monitoring.

(3) The analysis for social camps

According to the stability theorem of differential equations, to determine whether the strategy results of social camps is in a stable state, it is necessary to simultaneously satisfy F_SC_(z) = 0 and ∂F_SC_(z)/∂z<0, that is:

$$\frac{\partial F_{SC}(z)}{\partial z}=(2z-1)[C_{s}-R_{s}+(1-x){\left(R_{s}-C_{s}\right)}^{\alpha_{s}}+xR_{s}+yR_{s}-xyR_{s}]$$ (16)

Suppose that S(x) is a function of x.


$$S(x)=C_{s}-R_{s}+(1-x){\left(R_{s}-C_{s}\right)}^{\alpha_{s}}+xR_{s}+yR_{s}-xyR_{s}$$ (17)

$$x^{*}=\frac{{\left(R_{s}-C_{s}\right)}^{1-\alpha_{s}}+yR_{s}}{{\left(R_{s}-C_{s}\right)}^{\alpha_{s}}-R_{s}+yR_{s}}$$ (18)

**Fig 4:**
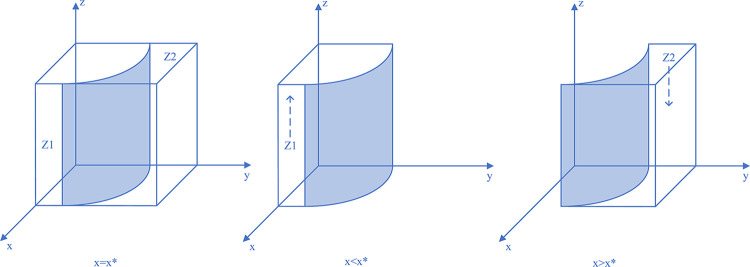
Social camps strategy replication dynamic phase diagram.

Thus, when x = x*, S(x) = 0, and ∂F_SC_(z)/∂z≡0, the system reaches a steady state regardless of the value of x and the social camps have no evolutionary stabilization strategy; if x<x*, S(x)<0, and ∂F_SC_(z)/∂z|_z = 0_<0, at which point z = 1, the evolutionary stabilization strategy of the social camps is proactive monitoring. Similarly, and conversely, at z = 0, the evolutionary stabilization strategy of the social camps is no monitoring. The replicated dynamic phase diagram of the social camps’ strategy is shown in Fig 4.

**Inference 5:** The probability of social camps actively supervising is positively correlated with the reporting reward and risk-pursuit coefficient, and negatively correlated with the cost of supervision. It follows that strengthening the environmental awareness of social camps, their awareness of dust pollution, and the credibility of central inspection institutions, protecting the privacy of whistleblowers, reducing the risk and cost of reporting, and increasing the perceived benefits of supervision strategies can promote the selection of active supervision strategies by social camps. This can effectively promote the implementation of dust pollution regulation through a coordinated supervision mode.

**Inference 6:** The execution of monitoring strategies by social camps is jointly affected by the strategies of coal mining enterprises and local regulators. When coal mining enterprises engage in illegal pollution discharge and local regulators neglect their duties, it is easy to breed the phenomenon of collusion between government and enterprises. Social camps tend to exercise their monitoring rights and report to the central inspection institutions to obtain rewards. When coal mining enterprises choose to actively engage in pollution control, social camps will evolve into the no monitoring strategy. Meanwhile, in a social environment where local regulators continue to implement strict regulated, regardless of the pollution control strategies chosen by coal mining enterprises, social camps will also evolve into the no monitoring strategy.

#### System stability analysis

Different equilibrium points exist in replicated dynamic systems. If the trajectory curve of a certain equilibrium point converges to that point when starting from any adjacent area, that equilibrium point is regarded as locally asymptotically stable. The combination of different equilibrium points corresponds to the Evolutionary Stable Strategy (ESS) [30]. By setting F_CM_(x) = F_LG_(y) = F_SC_(z) = 0, we obtain eight special equilibrium points in the replicator dynamics system, namely E_1_(0,0,0), E_2_(0,0,1), E3(0,1,0), E_4_(0,1,1), E_5_(1,0,0), E_6_(1,0,1), E_7_(1,1,0), and E_8_(1,1,1) as well as six mixed strategy equilibrium solutions. The evolutionary stable equilibrium of multi-agent evolutionary games is always a strict Nash equilibrium. Mixed strategy Nash equilibrium cannot withstand the accumulation of small disturbances over a long period of time, ultimately evolving into pure strategy Nash equilibrium. As such, we shall only discuss the asymptotic stability of the aforementioned eight special points [31]. The Jacobian matrix of a three-player game is represented by Formulas 19 and 20.


$$J=[\begin{array}{ccc} \frac{\partial F_{CM}(x)}{\partial x} & \frac{\partial F_{CM}(x)}{\partial y} & \frac{\partial F_{CM}(x)}{\partial z}\\ \frac{\partial F_{LG}(y)}{\partial x} & \frac{\partial F_{LG}(y)}{\partial y} & \frac{\partial F_{LG}(y)}{\partial z}\\ \frac{\partial F_{SC}(z)}{\partial x} & \frac{\partial F_{SC}(z)}{\partial y} & \frac{\partial F_{SC}(z)}{\partial z} \end{array}]$$ (19)

Of which,

$$\left\{ \begin{array}{l} \frac{\partial F_{CM}(x)}{\partial x}=(2x-1)\left\{\begin{array}{l} C_{m}-R_{m}-yE-yP_{m}-yS-(1-y)z(1-\gamma )E\\ +(y+z-yz-1)\lambda_{m}[(1-\gamma )E^{\beta_{m}}] \end{array}\right\}\\ \frac{\partial F_{CM}(x)}{\partial y}=-x(x-1)\{E+P_{m}+S-z(1-\gamma )E-(1-z)\lambda_{m}[(1-\gamma )E^{\beta_{m}}]\}\\ \frac{\partial F_{CM}(x)}{\partial z}=x(x-1)(y-1)\{\left(1-\gamma )E-\lambda_{m}[(1-\gamma )E^{\beta_{m}}\right]\}\\ \frac{\partial F_{LG}(y)}{\partial x}=y(y-1)[P_{m}+S-P_{m}{}^{\alpha_{l}}+z(1-\lambda_{l}){\left(\gamma E\right)}^{1+\beta_{l}}]\\ \frac{\partial F_{LG}(y)}{\partial y}=(2y-1)[\begin{array}{l} C_{l}-(1-x)P_{m}{}^{\alpha_{l}}+xS-\lambda_{l}{\left(\gamma E\right)}^{\beta_{l}}\\ -(1-x)z\gamma E+(1-x)z\lambda_{l}{\left(\gamma E\right)}^{\beta_{l}} \end{array}]\\ \frac{\partial F_{LG}(y)}{\partial z}=(x-1)y(y-1)[\gamma E-\lambda_{l}{\left(\gamma E\right)}^{\beta_{l}}]\\ \frac{\partial F_{SC}(z)}{\partial x}=z(z-1)[R_{s}-{\left(R_{s}-C_{s}\right)}^{\alpha_{s}}-yR_{s}]\\ \frac{\partial F_{SC}(z)}{\partial y}=-(x-1)z(z-1)R_{s}\\ \frac{\partial F_{SC}(z)}{\partial z}=(2z-1)[C_{s}-R_{s}+(1-x){\left(R_{s}-C_{s}\right)}^{\alpha_{s}}+xR_{s}+yR_{s}-xyR_{s}] \end{array}\right.$$ (20)

By substituting the eight special equilibrium points into the Jacobian matrix, we obtain the corresponding eigenvalues for each equilibrium point, as shown in Table 3. According to Lyapunov’s first method, the eigenvalues of the Jacobian matrix can be used as the criteria for determining the evolutionary stability. If all eigenvalues of the Jacobian matrix are less than 0, then it can be determined that the equilibrium point is an Evolutionary Stable Strategy (ESS). Conversely, if any one eigenvalue is positive, then the corresponding equilibrium point cannot be an ESS. Due to different parameter values, some equilibrium points have different signs of corresponding eigenvalues, implying the direction of system evolution is different. From the states of each point in Table 3, the {Active Governance, Negligence, Proactive Monitoring} and {Active Governance, Strict Regulation, No Monitoring} are potential evolutionarily stable strategies, which are the ideal states studied and require further discussion and research.

**Table 3:** Special equilibrium points and stability of evolutionary game models.

Equilibrium points	Jacobian matrix eigenvalues and symbols				Stability
	Λ1	Λ2	Λ3	Symbols	
**E**_**1**_ **(0,0,0)**	λ_m_[(1-γ)E]^β^_m_ +R_m_-C_m_	λ_l_(γE) ^β^_l_-P_m_^α^_l_ +P_m_-C_l_	(R_s_-C_s_)^1-α^_s_	(+/-,+/-,+)	Instability point
**E**_**2**_ **(0,0,1)**	(1-γ) E+R_m_ -C_m_	P_m_^1-α^_l_+γE-C_l_	(R_s_-Cs) ^α^_s_^-1^	(+/-,+/-,+)	Instability point
**E**_**3**_ **(0,1,0)**	E+P_m_+S+R_m_ -C_m_	λ_l_(γE) ^β^_l_+C_l_ -P_m_^1-α^_l_	-C_s_-(R_s_-C_s_) ^α^_s_	(+,+/-,-)	Instability point
**E**_**4**_ **(1,0,0)**	C_m_-R_m_-λ_m_[(1-γ)E]^β^_m_	λ_l_(γE) ^β^_l_ -C_l_ -S	-C_s_	(+/-,+/-,-)	Uncertainty
**E**_**5**_ **(1,1,0)**	C_m_-R_m_-P_m_-S -E	-λ_l_(γE) ^β^_l_ +C_l_ +S	-C_s_	(-,+/-,-)	Uncertainty
**E**_**6**_ **(1,0,1)**	C_m_-R_m_ -(1-γ) E	λ_l_(γE) ^β^_l_ -C_l_ -S	C_s_	(-,+/-,+)	Instability point
**E**_**7**_ **(0,1,1)**	E+P_m_+R_m_+S -C_m_	-P_m_^1-α^_l_-γE+C_l_	C_s_+(R_s_-C_s_) ^α^_s_	(+,+/-,+)	Instability point
**E**_**8**_ **(1,1,1)**	-E-P_m_-R_m_-S-+C_m_	-λ_l_(γE) ^β^_l_ +C_l_ +S	C_s_	(-,+/-,+)	Instability point

**Inference 7:** E_2_(0,0,1), E6(1,0,1) and E7(0,1,1) are unstable points. This indicates that when the social camps take the initiative to monitor dust pollution, effective reporting of supervisory measures will be taken by the central inspection agency against the polluting party. Neither the coal mining enterprises nor the local regulators will engage in illegal and non-compliant behavior. Thus, it is impossible to achieve an Evolutionarily Stable Strategy (ESS) that leads to the balance of ideal states in the system. This highlights the importance of the involvement of the social camps in the co-regulation model of dust pollution, which plays an important role in supervising and encouraging coal mining enterprises to actively manage pollution and local regulators to strictly enforce regulations.

**Inference 8:** If C_m_-R_m_<λ_m_[(1-γ)E]^β^_m_ and λ_l_(γE) ^β^<C_l_+S, then E_4_(1,0,0) is an Evolutionarily Stable Strategy (ESS). This indicates that when the pollution responsibility sharing coefficient is small, the environmental compensation cost of expected risk associated with illegal emissions by coal mining enterprises exceeds the cost and benefits of dust pollution control, and local regulators have a lower risk perception cost of strict regulation than the actual cost of regulation, the Evolutionarily Stable Strategy for the three agents is {Active Governance, Negligence, No Monitoring}. Under such circumstances, coal mining enterprises are required to implement strict self-control measures and actively manage dust pollution, which will lead to benefits in terms of subsidies and governance. In contrast, the local regulators and the social camps do not have to monitor the pollution situation, hence the local regulators’ expected risk cost is lower than the monitoring cost, resulting in regulatory negligence.

**Inference 9:** If λ_l_(γE) ^β^_l_ >C_l_+S, then E_5_(1,1,0) is an Evolutionarily Stable Strategy (ESS). This indicates that when the pollution responsibility sharing coefficient is large, the expected risk cost of illegal emissions by coal mining enterprises exceeds the cost and benefits of dust pollution control, and the risk perception cost of local regulators is greater than the actual cost of strict regulation, the Evolutionarily Stable Strategy for the three agents is {Active Governance, Strict Regulation, No Monitoring}. This implies that the coordinated dust pollution supervision model continues to be effective, making it difficult for coal mining enterprises and local regulators to conspire. Instead, they each perform their respective duties and jointly promote dust pollution control, without the need for additional monitoring by the social camps. Consequently, the ecological environmental quality of mining areas is significantly improved, representing the ideal state of the coordinated dust pollution governance model.

## Numerical simulation and discussion

### Parameters setting

In order to explore how parameter changes, affect game-theoretic behavior of agents, we used MATLAB software. We analyzed the relevant parameters and potential evolutionary game stable strategies in Inference 8 and 9. The parameter settings used in the game model are shown in Table 4, all the above parameters are pure numbers without units. In which, the E, C_m_, R_m_ and P_m_ parameters of coal mining enterprises are sourced from previous research [1–3, 5, 25, 32] and provided by the China National Coal Association(CNCA), the γ, S and C_l_ parameters of central and local regulatory authorities, the C_s_ and R_s_ parameters of social camps are referred to the previous research [6–8, 11, 13, 25, 33].The risk-seeking α, β and loss-aversion coefficients λ in the prospect theory formula were assigned values based on the research conducted by Tversky and Kahneman [34].

**Table 4:** Parameter value setting.

Parameter	γ	E	C_m_	R_m_	P_m_	S	C_l_	C_s_	R_s_	α_l_, α_s_	β_m_	β_l_	λ_m_	λ_l_
**Array1**	0.3	10	8	2	3	1	3	0.5	1.5	0.88	0.88	0.88	2.25	2.25
**Array2**	0.3	10	8	2	2	1	3	0.5	1.5	0.88	1	0.44	1.25	1.25

### Game factors simulation and discussion

Experiment 1: The effect of the responsibility sharing coefficient γ on the evolutionary game process and outcome.

**Fig 5:**
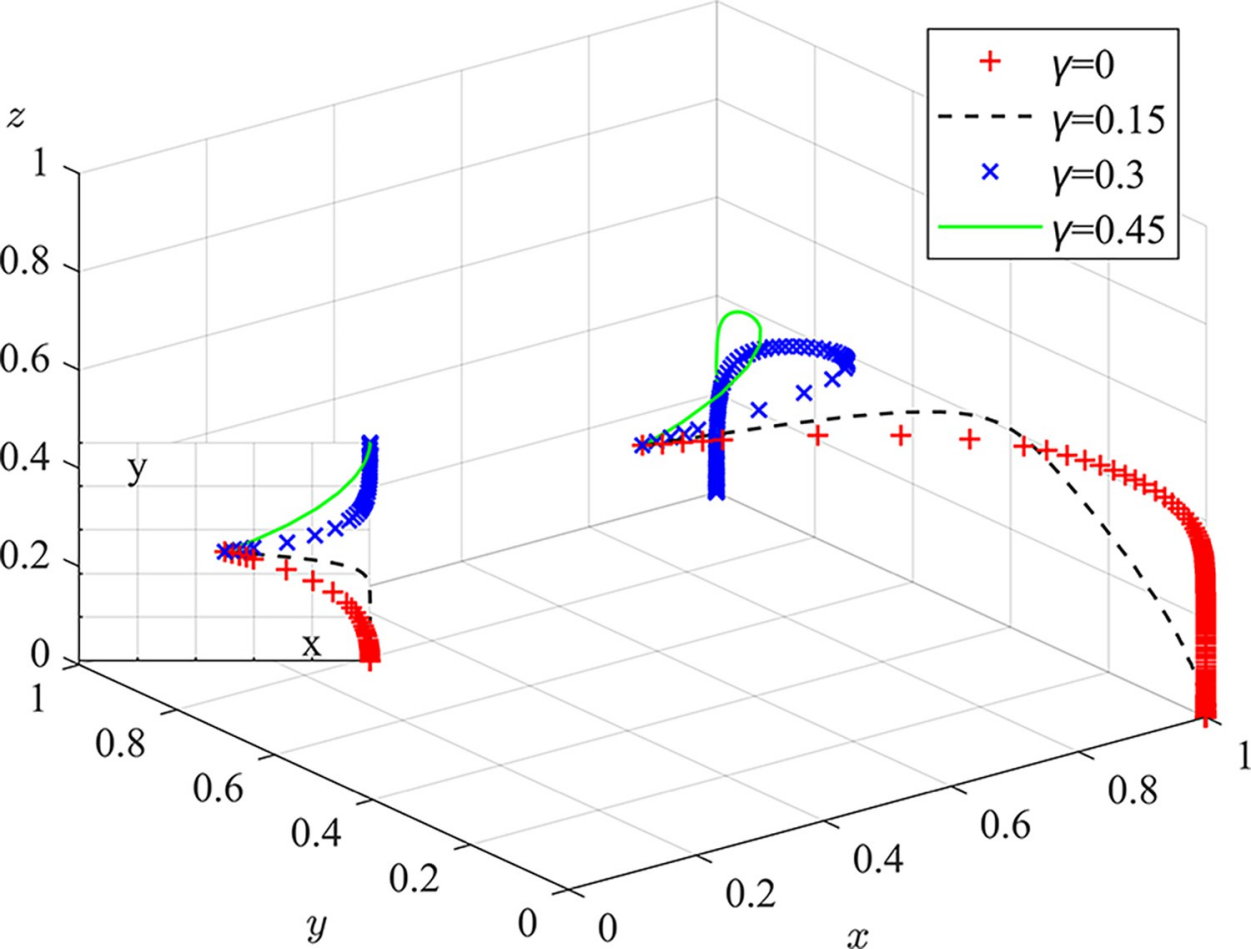
Impact of responsibility sharing coefficients on the evolutionary game process and outcome.

Based on the initial value condition, the responsibility sharing coefficient was set to 0/0.15/0.3/0.45, and the simulation results are shown in Fig 5. The experimental results indicate that a too low responsibility sharing coefficient contributes to local regulators neglecting their duties. With an increase responsibility sharing coefficient, the rate of system evolution accelerates, and the strategic choices of local regulators evolve gradually from misconduct to strict regulation. By formulating appropriate responsibility sharing coefficient, the central environmental protection department can increase the joint liability of local regulator for pollution penalties, enhance the punishment risk for regulatory dereliction, prompt the conscientiousness of local regulator employees, and ensure the stability and effectiveness of the coordinated governance model for coal dust pollution. At the same time, this approach can alleviate the pressure exerted by the central environmental protection department on local subordinate entities, reduce the problems of inadequate regulatory enforcement and poor information transmission caused by long chains of administrative management.

Experiment 2: The effect of pollution remediation expenditure E on the evolutionary game process and outcome.

**Fig 6:**
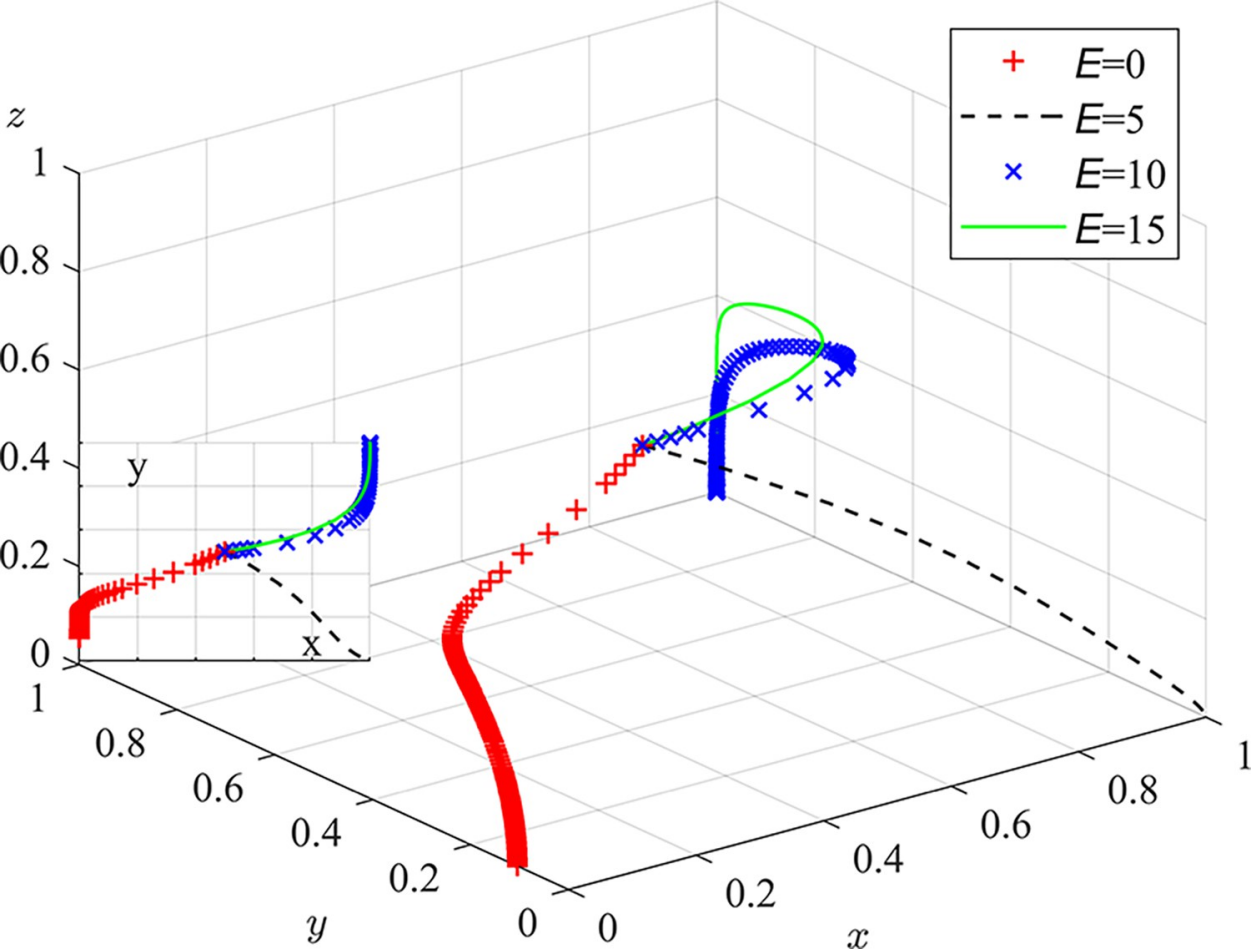
Impact of pollution remediation expenditures on the evolutionary game processes and outcome.

Based on the initial value condition, the pollution remediation delay loss is set to 0/5/10/15, the simulation results are shown in Fig 6. The experimental results show that when all pollution compensation expenditures are borne by the central environmental protection department, coal mine enterprises tend to choose illegal discharge, and the trend of strict regulation by local regulators is low. With an increase in pollution compensation expenditure, the rate of system evolution accelerates. In the beginning, coal mine enterprise’s strategic choices undergo a change, from illegal discharge to active governance. Immediately after, the strategic choices of local regulators undergo a change, from neglect of duty to strict regulation. Part of the pollution compensation expenditure is "virtual expenditure" to compensate for damage to public credibility. The weight of coal mine enterprises and local regulators’ evaluations include the effectiveness of dust control, environmental protection responsibilities, and the number of pneumoconiosis cases directly related to dust pollution. By using internet platforms to strengthen environmental protection and occupational disease publicity efforts, deepen the public’s concept of protecting the ecological environment and safeguarding legal rights, and increase the importance of public credibility to gaming subjects, it will help the coordinated governance model for coal dust pollution to operate smoothly.

Experiment 3: The effect of the cost of pollution control C_m_ and the additional benefit of pollution control R_m_ on the course and outcome of the evolutionary game.

**Fig 7:**
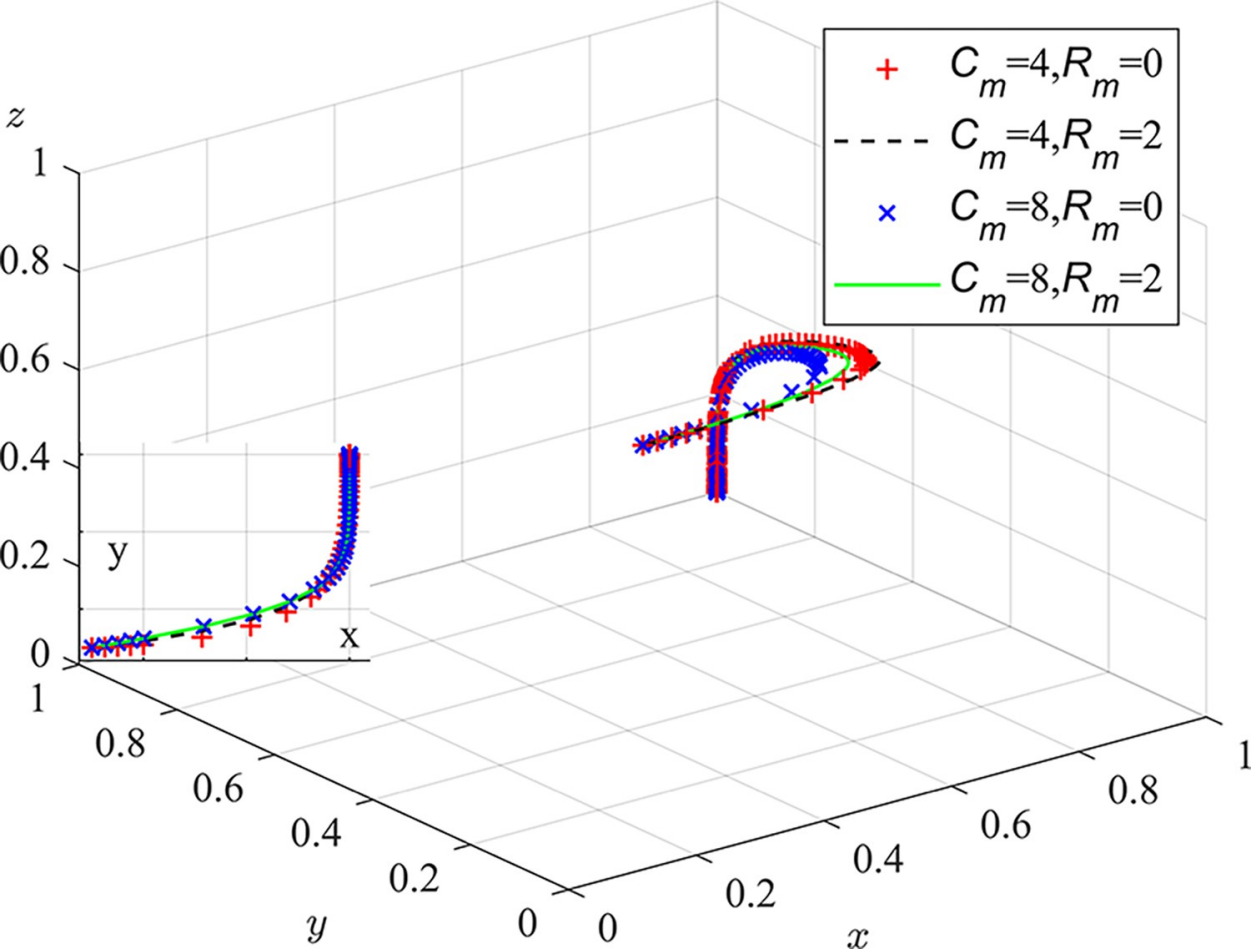
Impact of pollution control costs and abatement additional benefits on the evolutionary game process and outcome.

Based on the initial value condition, the cost of pollution control is set to 4/8 and the additional benefit of pollution control is set to 0/2. The simulation results are shown in Fig 7. The experimental results demonstrate that the greater the difference between pollution control costs and additional pollution control benefits, the faster the rate of system evolution. For a fixed pollution control cost, the smaller the additional pollution control benefits, the faster the rate of system evolution. At the same time, for a fixed additional pollution control benefit, the greater the pollution control cost, the faster the rate of system evolution. Coal mine enterprises should develop or introduce advanced dust control technologies, and comprehensively use methods such as chemical dust suppressants, windbreak dust suppression nets, closed dust suppression systems, dry and wet dust removal, green belts, polyester covering films, etc., in order to reduce pollution control costs and promote a green transformation of the industrial structure, ultimately enhancing the additional benefits of improved environmental quality and happiness resulting from the control of dust pollution. At the same time, local regulators should provide certain financial and tax support to related environmental protection industries, promote the research and development of dust pollution control technologies and the development of pollution control equipment, reduce the cost of pollution control materials and equipment technology, alleviate the pressure of coal mine enterprise’s investment in dust pollution control, and enhance their motivation and additional benefits of controlling pollution.

Experiment 4: The effect of the environmental penalty P_m_ on the course and outcome of the evolutionary game.

**Fig 8:**
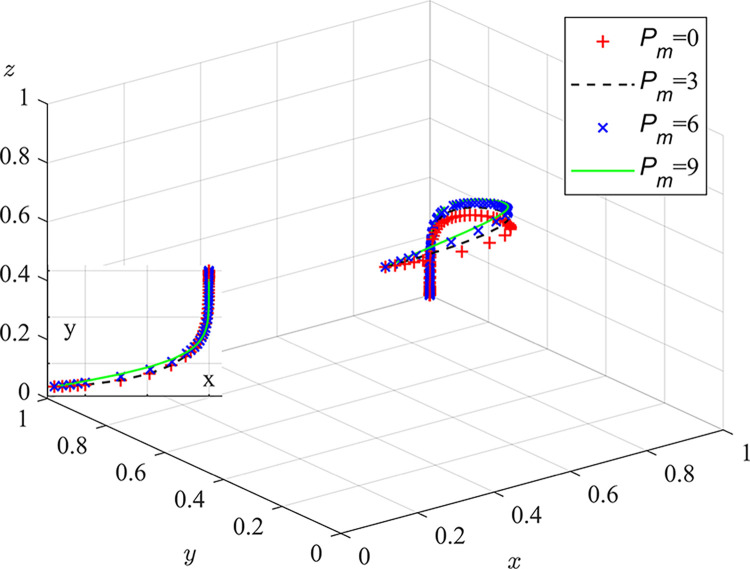
Impact of environmental fines on the evolutionary game process and outcome.

Based on the initial conditions, the environmental penalty was set to 0/3/6/9. The results of the simulation are shown in Fig 8. The experimental results indicate that as the environmental fine increases, the rate of system evolution accelerates. When no environmental fine is imposed, the strategic evolution of coal mine enterprises and local regulators tends toward an ideal outcome. In combination with Inference 7, it is believed that the introduction of social camps will mean that the illegal behavior of coal mine enterprises and local regulators will always be discovered and punished by central inspection agencies, making the imposition of environmental fines unnecessary to achieve optimal outcomes. Therefore, in the coordinated governance model for coal dust pollution, the influence of the number of environmental fines on the results of system evolution is minimal, and the imposition of fines is not an effective means of dust regulation. Local regulators’ environmental fine amounts should reference relevant documents from the national environmental protection department or similar areas’ punishment situations. High environmental fines will increase the financial pressures on coal mine enterprises operations and weaken the local business environment of coal mines to some extent.

Experiment 5: The effect of the pollution control subsidy S on the course and outcome of the evolutionary game.

**Fig 9:**
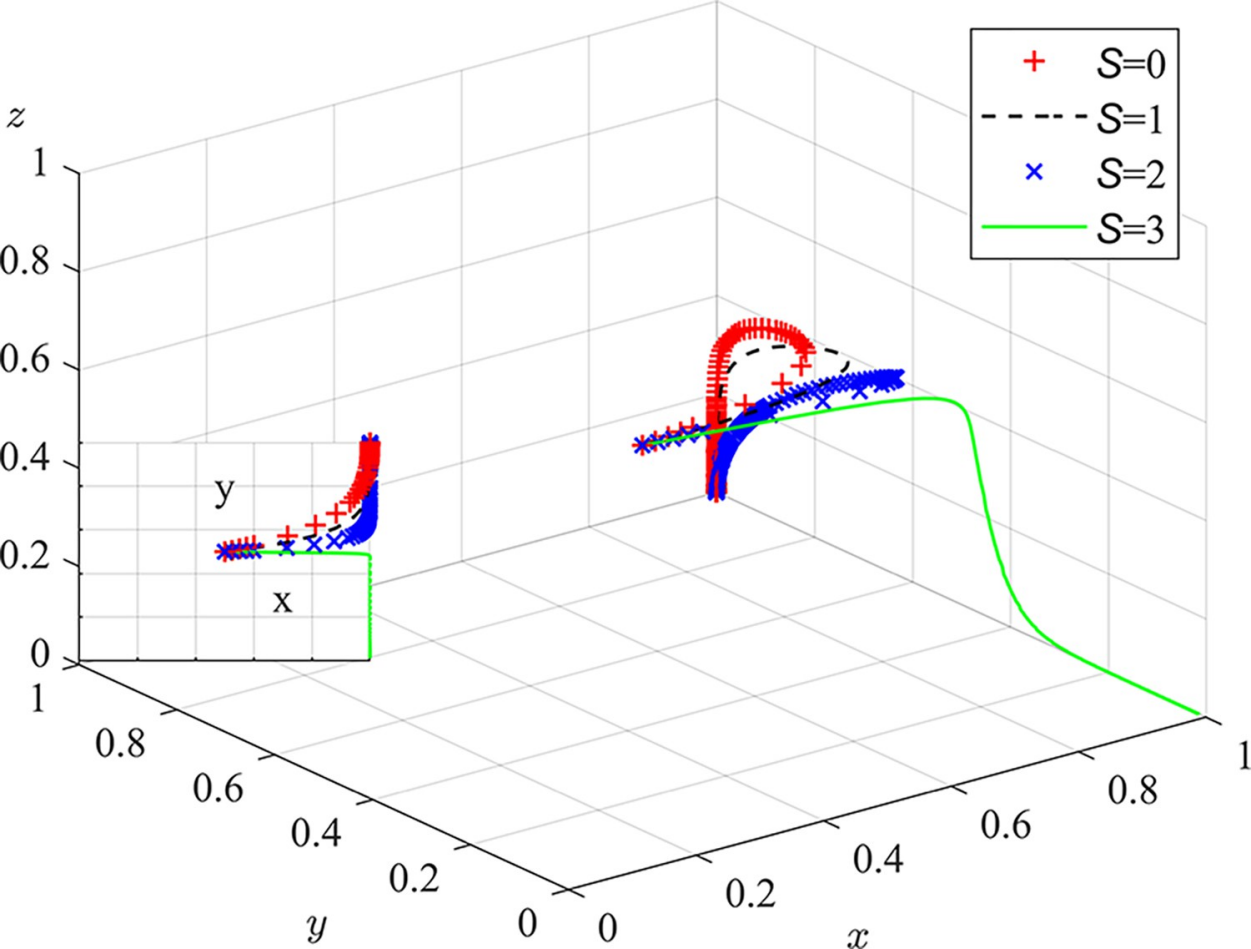
Impact of pollution control subsidies on the evolutionary game process and outcome.

Based on the initial conditions, the pollution control subsidy was set to 0/1/2/3. The results of the simulation are shown in Fig 9. The experimental results demonstrate that as pollution control subsidies increase, the rate of system evolution accelerates. However, excessive pollution control subsidies can cause local regulators to evolve from strict supervision to neglect of duty. This is because pollution control subsidies are funded by local regulators and are given to coal mine enterprises as a certain amount of pollution control subsidies in the initial stage of system evolution, which can effectively stimulate the enthusiasm of coal mine enterprises to control dust pollution. As the evolution process progresses, the level of dust pollution control in coal mine enterprises improves, the additional benefits of pollution control increase, and the dust pollution control cost gradually decreases, forming a virtuous cycle of "low cost and high benefit" for dust pollution control. At this point, local regulators should reduce or stop subsidies for dust pollution control, reduce government financial pressure, and avoid negative impacts on normal supervisory work caused by insufficient budgetary funds.

Experiment 6: The effect of the regulatory cost C_l_ on the course and outcome of the evolutionary game.

**Fig 10:**
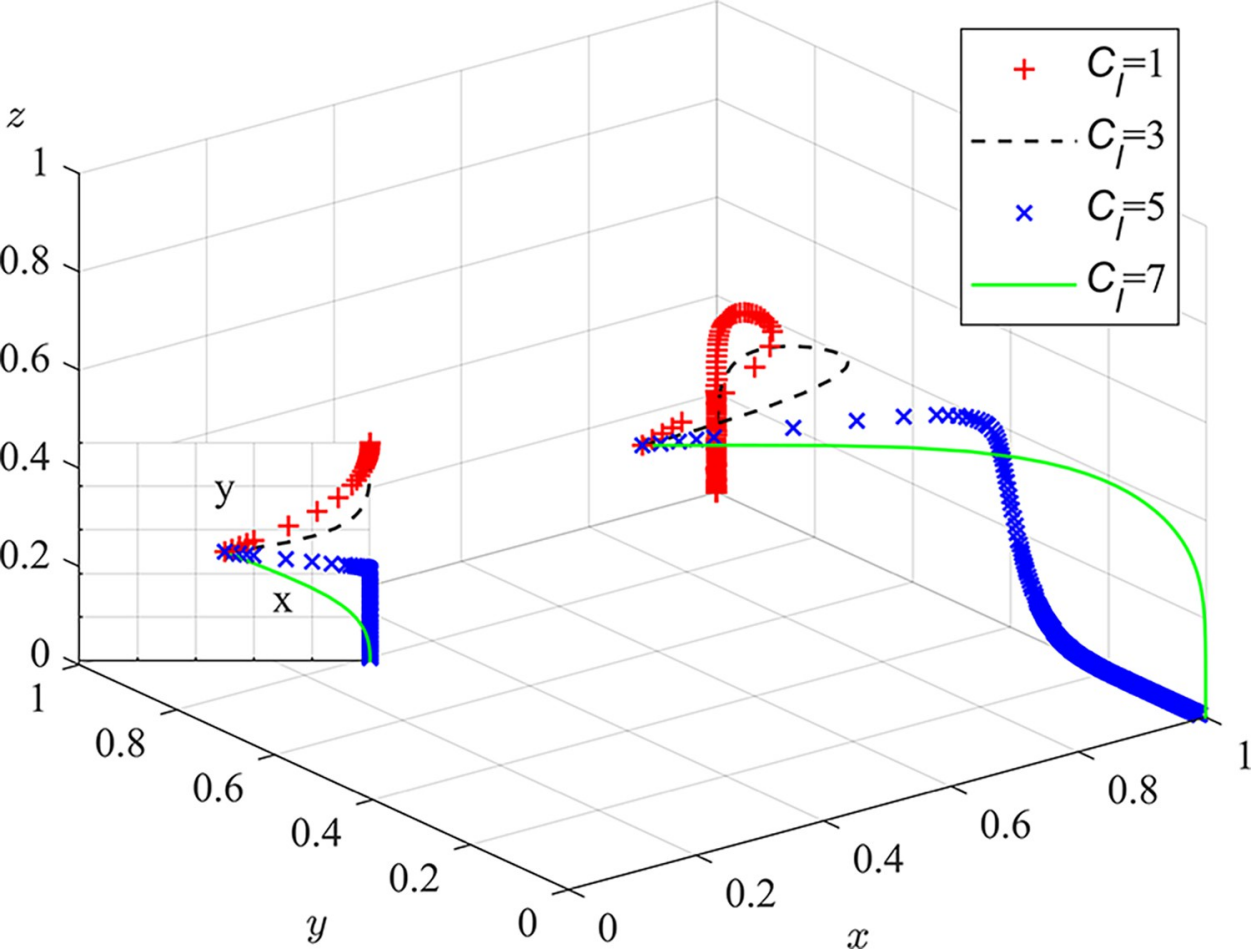
Impact of regulatory costs on the evolutionary game process and outcome.

Based on the initial conditions, the regulatory cost was set to 1/3/5/7. The results of the simulation are shown in Fig 10. The experimental results indicate that as regulatory costs increase, the rate of system evolution slows down. Excessive regulatory costs can lead to neglect of duty by local regulators. Local regulators can establish multiple dust monitoring stations and apply advanced pollution monitoring equipment and technologies such as drone monitoring and online monitoring systems to achieve online monitoring of the effect of dust pollution control measures in coal mine enterprises, timely grasp the full range of dust conditions, and improve monitoring efficiency. AI-based intelligent control technology, which relies on core technologies such as artificial intelligence and big data, can reduce on-site costs such as labor and staffing, reduce regulatory costs, and achieve intelligent pollution control practices to achieve a sustainable effect in dust pollution control. Digital technology can determine the location of pollution sources through GIS technology and apply targeted governance measures to the surrounding areas of pollution sources.

Experiment 7: The effect of coal mining enterprises’ risk-loss preferences λ_m_ and β_m_ on the process and outcome of the evolutionary game.

**Fig 11:**
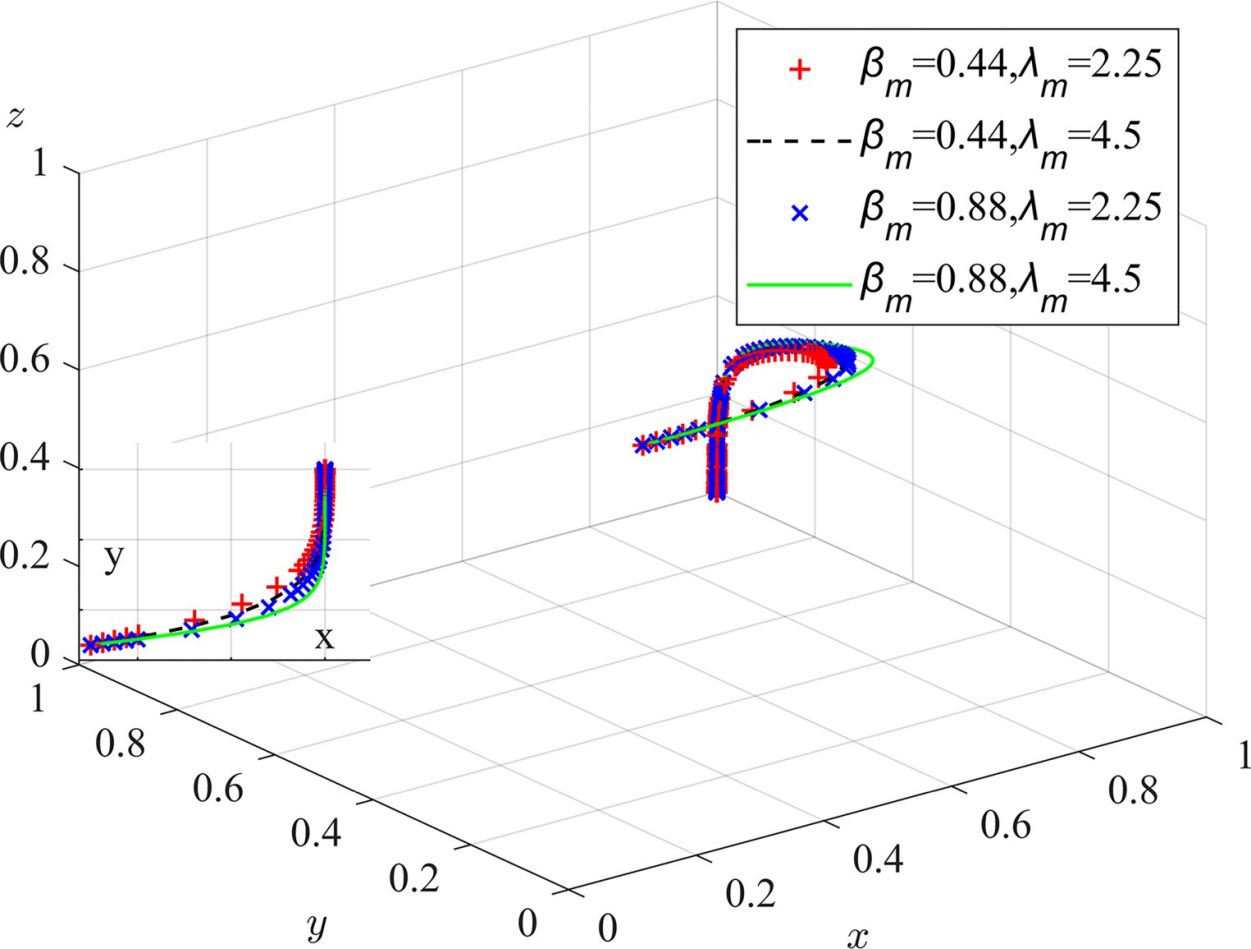
Impact of coal mining enterprises’ loss risk preferences on the evolutionary game process and outcome.

Based on the initial value conditions, the risk-loss attitude coefficient was set to 0.44/0.88 and the loss-avoidance coefficient responsibility sharing coefficient was set to 2.25/4.5. The results of the simulation are shown in Fig 11. The experimental results show that as the risk attitude coefficient, the loss avoidance coefficient, and the responsibility sharing coefficient increase, the rate of system evolution becomes faster. Coal mine enterprises, which prefer risk loss, need to pay attention to their pollution risk assessment capabilities. They can hire relevant experts to optimize risk assessment systems, improve the perception value of pollution and sensitivity to risk losses of coal mine enterprises, and strengthen the awareness of pollution control and the implementation of dust pollution control measures. The establishment of a sound occupational health management system can improve the dust prevention and control awareness of all employees in coal mine enterprises. A centralized occupational health education and training program centered on dust hazards should be conducted, focusing on monitoring and testing dust sources, providing personal protective equipment and conducting regular occupational hazard health check-ups to ensure the physical and mental health of employees[32].

Experiment 8: The effect of local regulators’ risk preferences α_l_, β_l_ and λ_l_ on the process and outcome of the evolutionary game.

**Fig 12:**
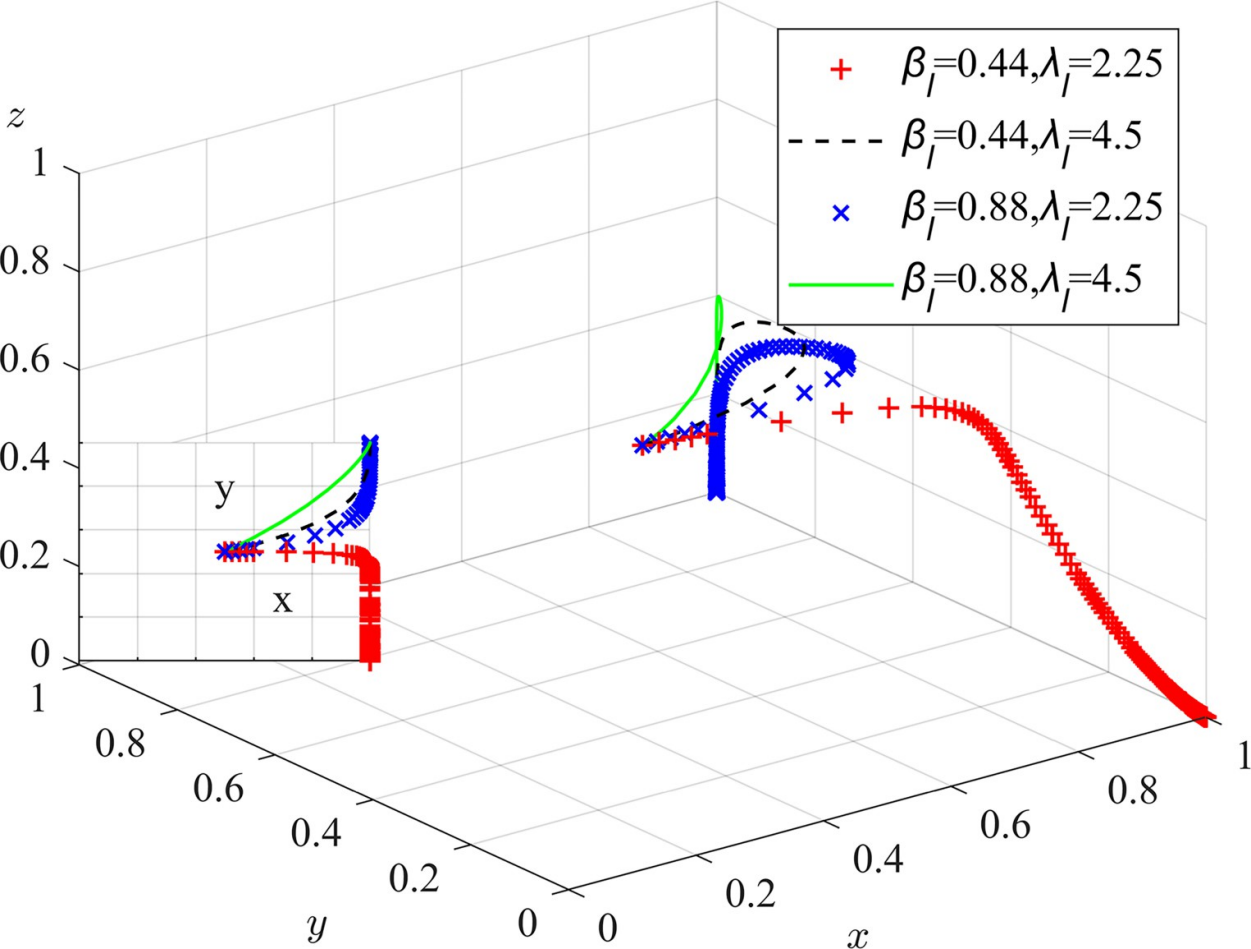
Impact of local regulators’ risk/reward preferences on the evolutionary game process and outcome.

**Fig 13:**
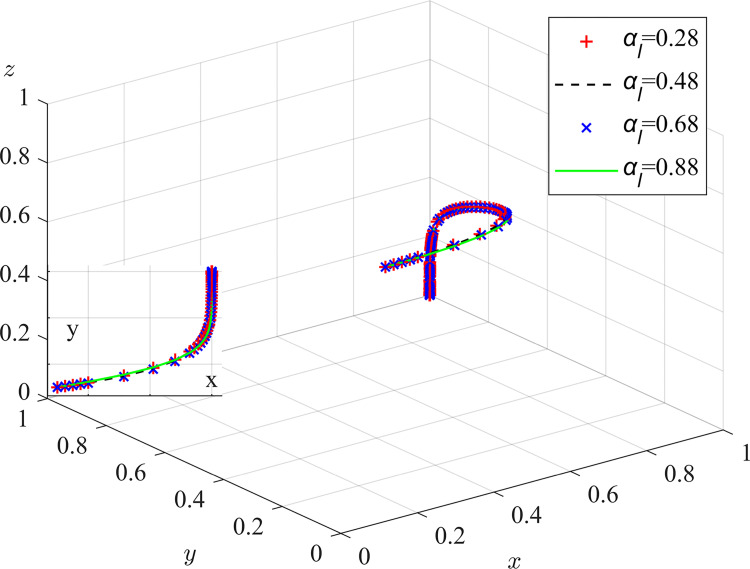
Impact of local regulators’ risk-loss preferences on the evolutionary game process and outcome.

Based on the initial value conditions, the risk-profit attitude coefficient was set to 0.11/0.22/0.44/0.88, the risk-loss attitude coefficient was set to 0.44/0.88, and the loss aversion responsibility sharing coefficient was set to 2.25/4.5. The simulation results are shown in Figs 12 and 13. The experimental results show that as the risk attitude coefficient and loss avoidance coefficient increase, the rate of system evolution becomes faster. The risk-profit attitude coefficient has a smaller impact on system evolution. Low perception of risk losses can lead to neglect of duty by local regulators. As a government agency, local regulators must comply with national regulations and prohibit the use of environmental fines for additional profits. Therefore, they are not sensitive to risk profits, but prefer risk losses. The responsibility and punishment from the central inspection department and the loss of credibility can have a negative impact on the normal revenue of local regulators. Regular special meetings should be held to emphasize the prevention of laziness and bottom-line thinking in risk management, enhance the risk preference, pollution-related punishment, neglect of duty exposure risk perception value and sensitivity to the loss of credibility of local regulators, urge them to perform their duties and strictly regulate dust pollution control measures.

### Game system simulation and discussion

**Fig 14:**
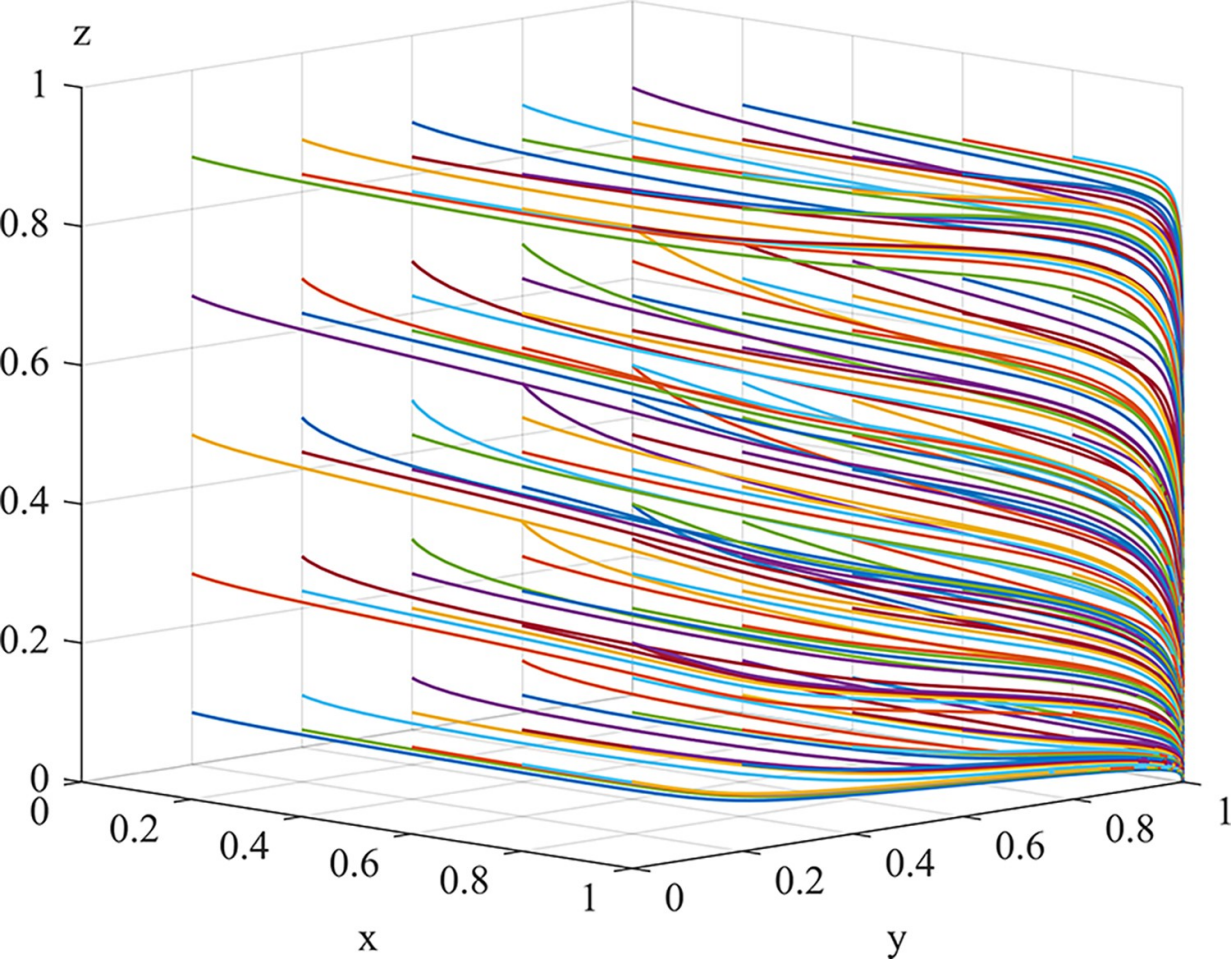
Simulation results for array 1(1,1,0) system.

Array 1 satisfies the conditions stated in Inference 9. It can be observed from Fig 14 that the strategies of coal mining enterprises and local regulators converge to 1, while the monitoring strategy of social camps converge to 0. Experimental results indicate that, regardless of the initial strategy combination, the ideal state of synergistic governance of dust pollution is achieved through active dust control by coal mining enterprises and strict supervision by local regulators, resulting in remarkable treatment effects. Although social camps have not been actively motivated to monitoring, their involvement increases the exposure and punishment risk of dust pollution and regulatory negligence. The high probability of active supervision accelerates system evolution and exerts a positive incentive effect on the active dust control by coal mining enterprises and strict supervision by local regulators. The experimental findings demonstrate that regardless of the initial strategy combination, a favorable scenario of proactive governance by coal mining enterprises and stringent oversight by local regulators is established, leading to effective coal mine pollution management, which represents the optimal state for the collaborative coal mine pollution management model. Although the social camp’s active monitoring behavior is not stimulated, its growth is critical, as it increases the risk of pollution failure and regulatory accountability. Moreover, the high likelihood of active monitoring accelerates the system’s evolution rate, which exerts a constraining effect on the active governance of coal mining enterprises and the strict regulation of local regulators.

**Fig 15:**
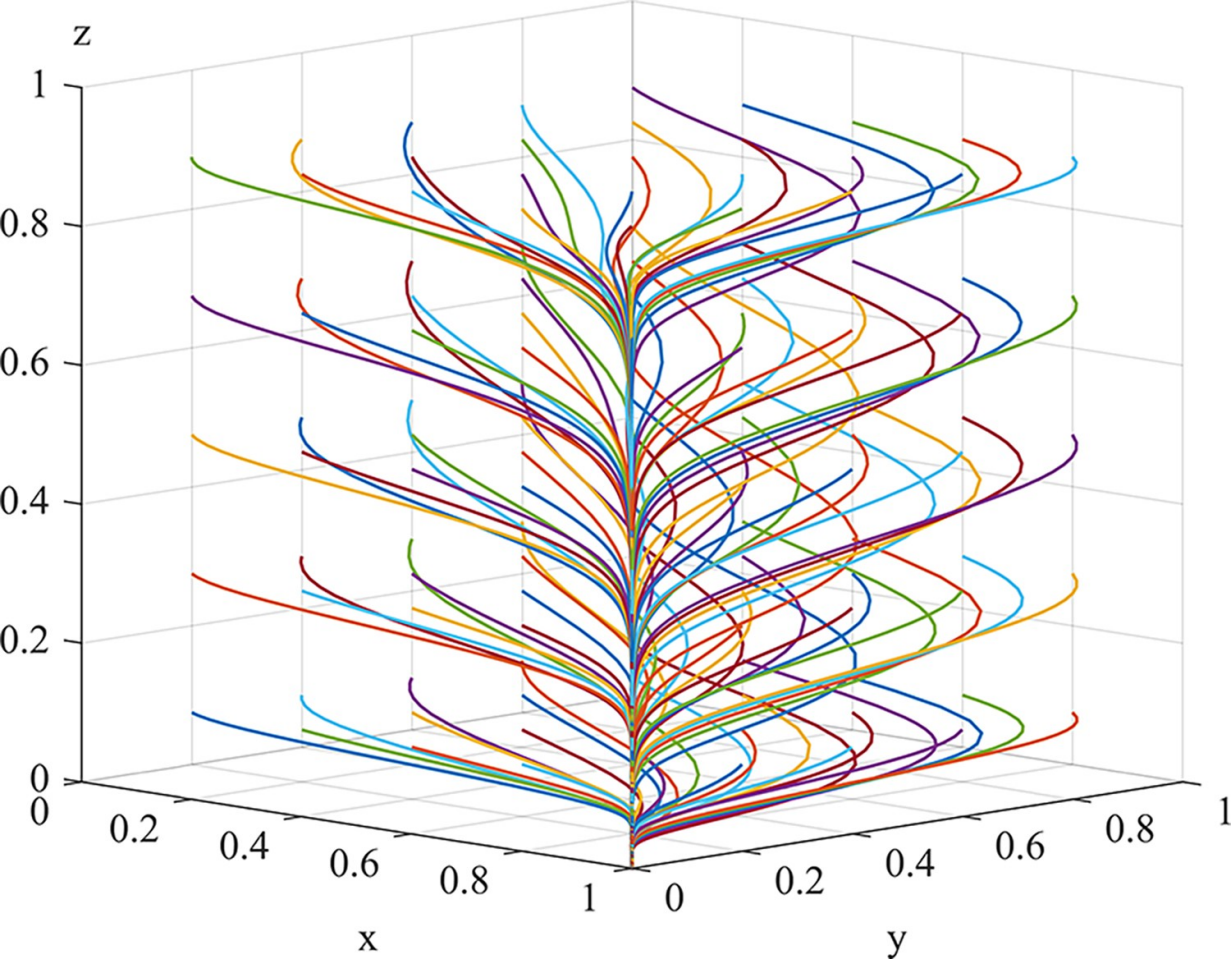
Simulation results for the array 2(1,0,0) system.

Array 2 satisfies the conditions stated in Inference 8, with local regulators exhibiting risk aversion. As can be observed from Fig 15, the strategy of coal mining enterprises converges to 1, while the supervisory strategies of both local regulators and social camps converge to 0. Experimental results indicate that, regardless of the initial strategy combination, coal mining enterprises are actively engaged in dust control, while local regulators tend to neglect their duties. This equilibrium differs from that of Array 1, as the involvement of social camps results in the partial division of the pollution inspection authority, originally held by local regulators. Given the regulatory cost that local regulators incur, if expected pollution risk losses and joint punishment losses are low, issues of neglect may arise. Corresponding policies are required to optimize this situation and restrain local regulators from neglecting their duties.

Thus, it can be seen, simulation analysis and the stability analysis of strategies by all agents are consistent and valid, providing practical guidance for the synergistic governance of dust pollution in opencast coal mines.

## Conclusion and implication

### Conclusion

Against the backdrop of the carbon peaking and carbon neutrality goals and the deepening concept of green and sustainable development, environmental governance has become a hot topic of research. In China, the problem of dust pollution in opencast coal mines has been unresolved for a long time, and the current national environmental governance system also has many shortcomings. Considering the reality of China, based on evolutionary game method combined with prospect theory, this study established a tripartite evolution game model analysis and simulation of coal mining enterprises, local regulators, and social camps, analyzed the factors and evolutionary paths that affect the participation of various stakeholders in dust pollution control strategies under different equilibrium conditions and parameter values. Therefore, the development laws and characteristics of the collaborative regulatory model of environmental governance can be more clearly presented and revealed. Moreover, this study introduces the psychological factor analysis of risk taking in prospect theory, improves the bounded rationality assumption in traditional evolutionary game theory, and enhances the application of evolutionary game theory in environmental governance. Additionally, this study contributes to improving the effectiveness of dust pollution control and promoting the innovative reform of China’s environmental governance system, and provides a useful reference for guiding the decision-making process of green and sustainable development of opencast coal mining enterprises. In addition, this study provides a practical theoretical basis for promoting China’s traditional energy transformation and creating social governance models, and has important practical significance and application value for China’s green development and ecological civilization construction.

Overall, this study strengthens the idea that the decision-making behaviors of the three parties in the game is influenced by each other, and the joint supervision of the social camps will urge coal mining enterprises to actively control pollution and local regulators to fulfil their duties. Furthermore, increasing joint liability for environmental pollution is beneficial for local regulatory authorities to strictly regulate environmental pollution, reduce the occurrence of collusion between officials and enterprises, and improve the effectiveness of environmental supervision. Meanwhile, the reduction of operating costs and the increase in associated benefits and rewards are conducive to the rapid and stable operation of the co-regulation model.

At the same time, this study also found some interesting differences from previous studies. The perceived value of dust pollution can have a significant impact on the strategic choices of the three parties in the game. Coal mining enterprises exhibit a preference for risk-taking. Considering prevailing punitive measures for pollution and associated social stigma, exposure to the risks posed by dust pollution entails high losses. As such, active governance measures become the optimal approach for coal mining enterprises. Similarly, local regulators also exhibit a preference for risk-taking, yet remain insensitive to risks and rewards. The environmental fine system has failed to sufficiently constrain dust pollution by opencast coal mining enterprises. On the other hand, governance subsidies have been an effective tool for incentivizing dust control, but excessively high subsidies can result in financial pressure on the government, as well as dilute the motivation for strict governance.

### Implication

The simulation results of this study have important managerial and practical implications. The conclusions of this study have certain implications for improving China’s environmental governance system, strengthening dust pollution control, and upgrading coal mining enterprises, as follows:

(1) The central environmental inspection agency can increase the joint punishment for local regulators that fail in their duties by reasonably raising the responsibility sharing coefficient. This would urge both coal mining enterprises and local regulators to act on dust pollution control and perform their duties. Increase public awareness of environmental laws and regulations, such as the Environmental Protection Law of the People’s Republic of China and the Air Pollution Prevention and Control Law, and promote popularization of environmental law and regulations. Set up convenient and efficient platforms such as the internet, WeChat public accounts, Weibo, and self-media platforms to report and disclose information on dust pollution. This would lower the cost of complaint and appeal for social stakeholders, allowing them to play a better supervisory role in controlling dust pollution and assisting local regulators. Clarify the legal boundaries of environmental disputes and promote socialization of dust pollution control. This would facilitate a multi-faceted transformation and upgrading of the new era’s social governance concept, model, tools, and objectives.
(2) Opencast coal mining enterprises should deeply implement the concept of green development and transform their dust control ideas from passive to active, enhancing environmental awareness and social responsibility. With the goal of improving efficiency, reducing costs, and reducing pollution and carbon emissions, advanced dust pollution control technologies should be comprehensively adopted to reduce dust pollution control costs, increase additional benefits of dust pollution control, and achieve a benign profit cycle of "small investment, large returns" for dust pollution control. This will become the core competitiveness of enterprises and achieve sustainable, high-quality development.
(3) Local regulators can encourage technological innovation in dust pollution control through fiscal and tax policies by adjusting coal mining resource taxes and reducing or exempting corporate income taxes for large-scale technological transformation projects. This will provide certain tax relief and cost support for technology R&D in dust pollution control. The use of dynamic pollution subsidy policies can optimize regulatory strategies for coal mining enterprises. In line with national development strategies, promoting the construction of ecological civilization should be an important criterion for performance evaluation and selection of staff in local regulators, with specific details defined and clarified.
(4) The establishment of legal dust environmental protection organizations can bring together stakeholders involved in dust pollution control, such as the general public, environmental organizations, and research institutions, to create a service platform that focuses on dust pollution control for opencast coal mining enterprises. The platform can provide publicity and education on the impact of dust pollution, raising environmental awareness, monitoring, and rights protection among society, making dust pollution control in coal mining enterprises more democratic. The adoption of the approach to industry-research cooperation can provide technical and policy services to opencast coal mining enterprises through environmental service outsourcing, assisting in their dust pollution control efforts. This approach also expands China’s environmental pollution control system.

### Deficiency and future prospect

Although this study has reached some conclusions of some theoretical and practical value, there are still some shortcomings that need to be further explored due to the complexity of the research issues. In the future, additional study can be done as follows:

On the one hand, the co-regulation model still needs to be supplemented in the construction of evolutionary game theory, and the design of the system parameters may not be comprehensive enough. The co-regulation model for dust pollution control in opencast coal mines involves many participants, such as central regulatory authorities, scientific research institutions, third-party governance institutions, etc., which will have an impact on the effectiveness of pollution control. In this study, only core entities from coal mining companies, local regulatory authorities and social camps are selected for research. At the later stage, external entities such as scientific research institutions may be included in the game system. Moreover, scholars will be able to analyses the collaborative governance models of different regions through actual case studies on the ground, and compare the differences and regional synergies between regions.

On the other hand, based on the digital economy era, the design of the parameters in the evolutionary game model may not be comprehensive enough in this study, and the application of digital technology in the model may not be fully considered. The application of digital technologies such as artificial intelligence and machine learning enables computers to independently simulate game processes and generate strategies using statistical methods to obtain probability distributions. In addition, data analysis techniques can be used to understand player behaviors, decision-making patterns, and strategies, and to generate predictions of strategies based on this. Data analysis techniques are also used to formulate new game rules, evaluate the safety and reliability of game objects, and other aspects. These applications make evolutionary game research and the construction of col-regulation model more scientific and rigorous [35]. Digital technology is widely used in pollution control, from monitoring, data analysis and waste management to source location, treatment design and environmental monitoring, and thus determine the optimal management plan. These applications help protect human health and environmental ecosystems, while supporting technological innovation and social development. Therefore, in follow-up studies, digital technologies such as big data and machine learning can be considered for parameter selection and simulation to open the black box of collaborative treatment models for opencast coal mine dust pollution from a more comprehensive perspective.
